# Movements and behaviour of blue whales satellite tagged in an Australian upwelling system

**DOI:** 10.1038/s41598-020-78143-2

**Published:** 2020-12-03

**Authors:** Luciana M. Mӧller, Catherine R. M. Attard, Kerstin Bilgmann, Virginia Andrews-Goff, Ian Jonsen, David Paton, Michael C. Double

**Affiliations:** 1 Cetacean Ecology Behaviour and Evolution Lab, Flinders University of South Australia, Adelaide, SA Australia; 2 Department of Biological Sciences, Macquarie University, Sydney, NSW Australia; 3 Australian Marine Mammal Centre, Australian Antarctic Division, Hobart, TAS Australia; 4 Blue Planet Marine, Canberra, ACT Australia

**Keywords:** Ecology, Zoology

## Abstract

Knowledge about the movement ecology of endangered species is needed to identify biologically important areas and the spatio-temporal scale of potential human impacts on species. Blue whales (*Balaenoptera musculus*) are endangered due to twentieth century whaling and currently threatened by human activities. In Australia, they feed in the Great Southern Australian Coastal Upwelling System (GSACUS) during the austral summer. We investigate their movements, occupancy, behaviour, and environmental drivers to inform conservation management. Thirteen whales were satellite tagged, biopsy sampled and photo-identified in 2015. All were genetically confirmed to be of the pygmy subspecies (*B. m. brevicauda)*. In the GSACUS, whales spent most of their time over the continental shelf and likely foraging in association with several seascape variables (sea surface temperature variability, depth, wind speed, sea surface height anomaly, and chlorophyll *a*). When whales left the region, they migrated west and then north along the Australian coast until they reached West Timor and Indonesia, where their movements indicated breeding or foraging behaviour. These results highlight the importance of the GSACUS as a foraging ground for pygmy blue whales inhabiting the eastern Indian Ocean and indicate the whales’ migratory route to proposed breeding grounds off Indonesia. Information about the spatio-temporal scale of potential human impacts can now be used to protect this little-known subspecies of blue whale.

## Introduction

An understanding about the movement ecology of wildlife is crucial for their management and conservation^1^, particularly for wide-ranging, endangered species. Information on the type and characteristics of movements, including occupancy patterns and behaviour, are needed to identify important habitats for these species, and the spatial and temporal scales at which human activities may impact upon them^1,2^. Advances in technological and analytical approaches for tracking movements and modelling behaviour of wildlife now makes it possible to gather this type of information, e.g.^3–6^. Yet there is still little movement data available for various species and populations^2^, including for many baleen whales.

Among baleen whales, blue whales (*Balaenoptera musculus*) are endangered due to twentieth century whaling, with different subspecies impacted at various levels^7,8^. Two of the currently recognised subspecies inhabit waters of the Southern Hemisphere and differ in morphology, vocal repertoire and geographic distribution^8–10^. The critically endangered Antarctic blue whale (International Union for the Conservation of Nature, IUCN; *B. m. intermedia*) feeds in Antarctic circumpolar waters during the austral summer, and likely breeds in lower latitudes of the Indian and Pacific Oceans, and perhaps also the Atlantic Ocean^8,9^, during the winter months. The data deficient pygmy blue whale (IUCN; *B. m. brevicauda*) feeds in temperate and subtropical waters in the austral summer, and presumably breeds in low latitudes of the Indian and western Pacific Oceans during the winter. The pygmy blue whale appears to be comprised of at least three acoustically and genetically differentiated populations: Indo-Australian, New Zealand, and Madagascar^9,11–16^.

While whaling is currently not a threat for blue whales, human activities on the marine environment could slow the recovery of their populations. Oil and gas exploration, shipping and fishing, as well as climate change, can disrupt blue whale behaviour, modify or displace whales from habitat, change prey availability, and cause physical injury that may lead to death, e.g.^17–20^. If impacts occur, whales are possibly at greater risk when they are in Biological Important Areas (BIAs). These are spatially defined areas where aggregations of individuals of a regionally significant species are known to display biologically important behaviours, such as feeding, calving and nursing, and where they may stay for prolonged periods of time. Although blue whales are the largest extant animals on Earth, there is still incomplete information about their migratory routes, feeding grounds, and breeding locations, particularly for southern hemisphere subspecies.

**Figure 1:**
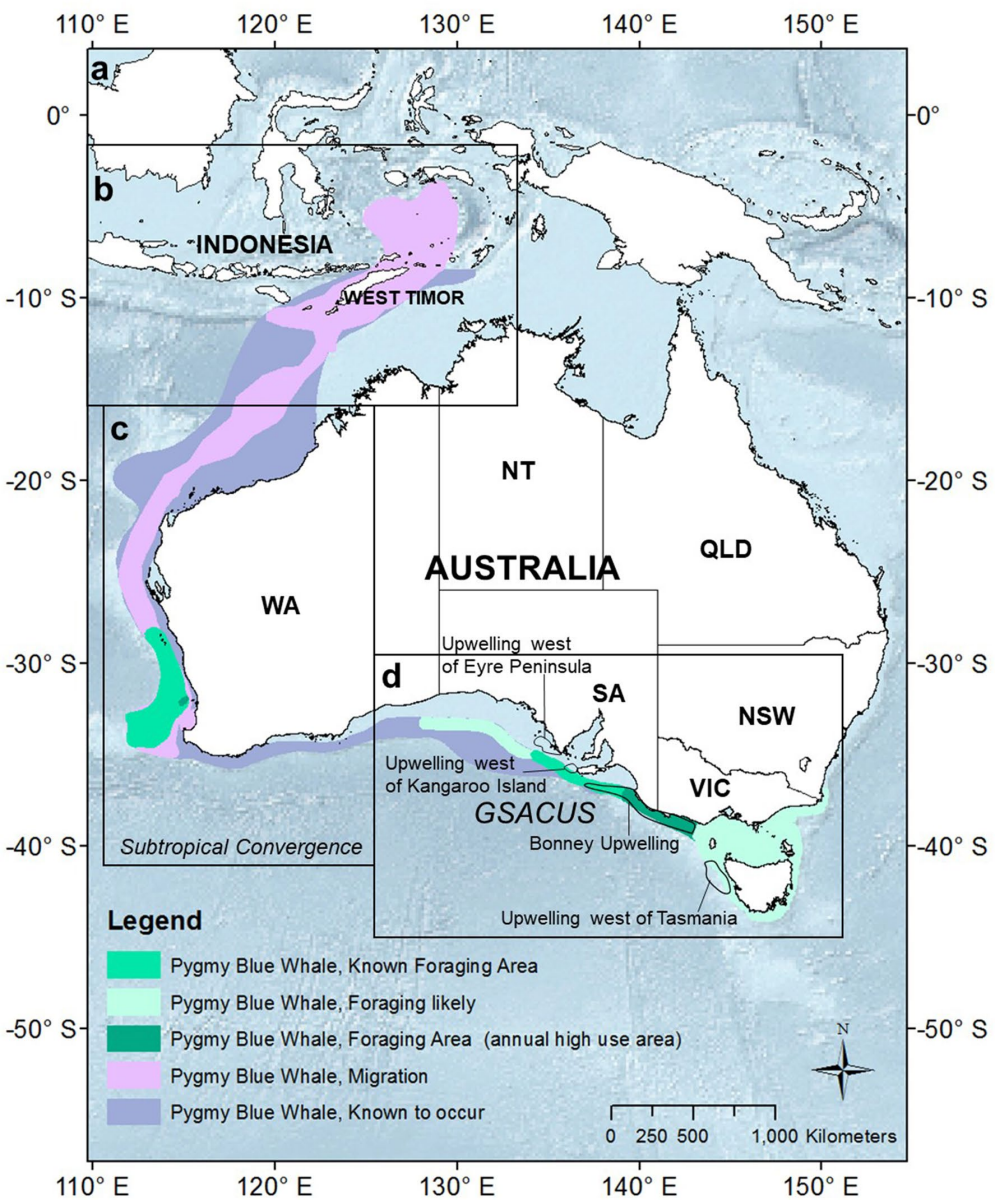

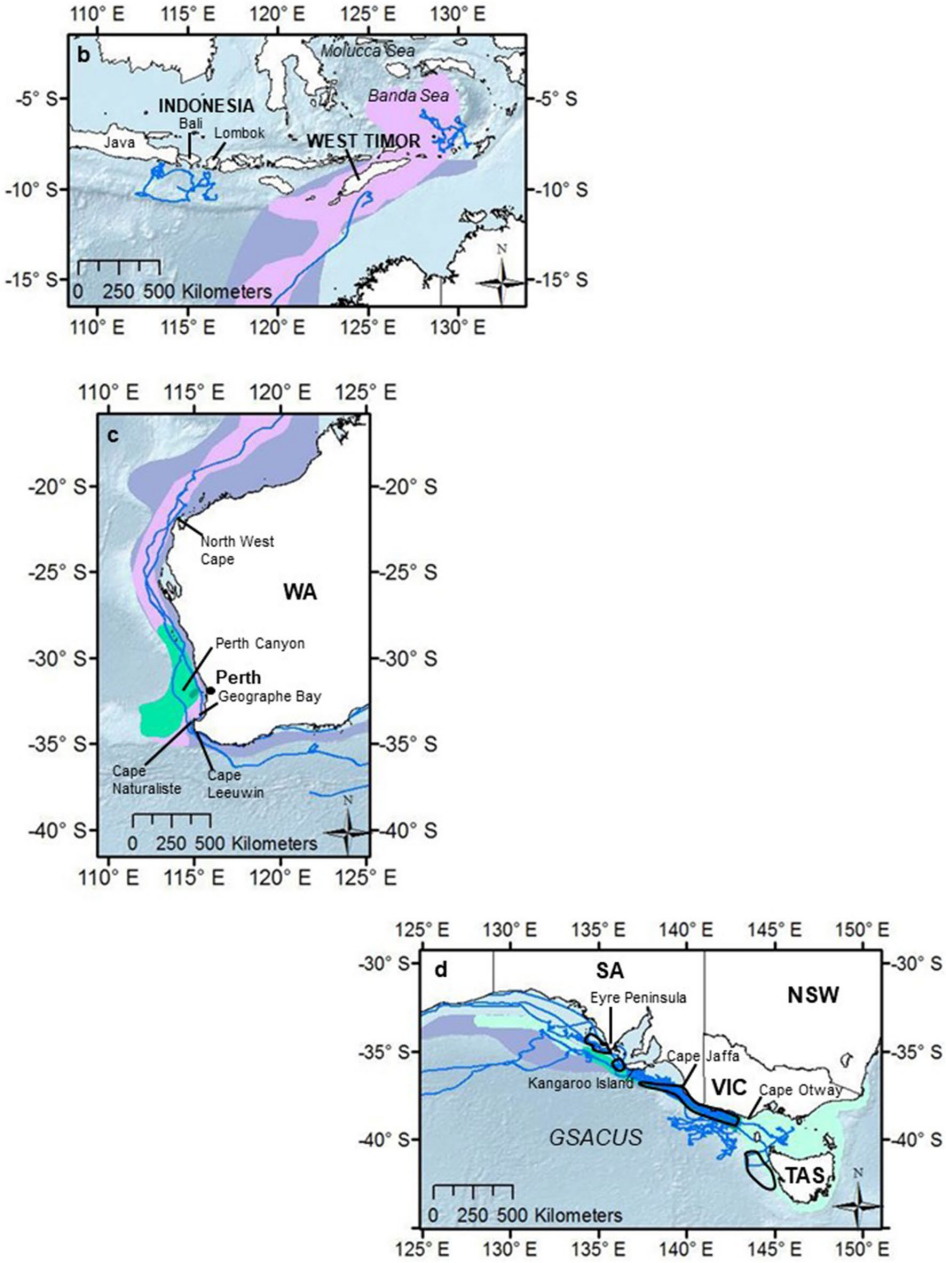
(**a**) Biological Important Areas (BIAs) for pygmy blue whales (*Balaenoptera musculus brevicauda*) as identified in the National Conservation Values Atlas of Australia^24^. Hierarchical switching state-space model (hSSSM) derived locations of 13 individuals satellite tagged in the Bonney Upwelling region, Great Southern Australian Coastal Upwelling System, southern Australia, with close-ups of (**b**) Indonesia, West Timor and northwestern Australia, (**c**) western and southwestern Australia, and (**d**) southern Australia. *WA* Western Australia; *SA* South Australia; *VIC* Victoria, *NSW* New South Wales, *TAS* Tasmania. Maps created in ArcGIS v.10 ( available at https://www.esri.com/).

In Australia, field studies have revealed two main feeding areas for blue whales—particularly, pygmy blue whales—where they can be observed between late spring to autumn each year. One area is the Perth Canyon, Western Australia (WA) (Fig. 1), where whales are normally sighted between March and May, and are believed to descend to depths of more than 300 m to feed on the krill *Euphasia recurva*^21^. The other known Australian feeding aggregation is in the Bonney Upwelling region, which forms off southern Australia from approximately Robe in South Australia (SA) to Portland in Victoria (VIC)^22,23^ each year (Fig. 1). In this region, blue whales can be sighted during the upwelling season between November and May, and have been observed surface feeding on swarms of the neritic krill *Nyctiphanes australis*^22,23^. Blue whale presence in the Bonney Upwelling is associated with several seascape variables, but with sea surface temperature appearing to play a major role^23^. Blue whales are also known to travel southward through Geographe Bay, southwestern WA, early in the season at around November and December^8^ (Fig. 1). Links between all these areas have been supported by some re-sightings of photo-identified blue whales between Perth Canyon, Bonney Upwelling and Geographe Bay^23^.

Satellite telemetry studies of eleven blue whales at the Perth Canyon have shown movements from the Perth Canyon area to the Naturaliste Plateau off south-west Australia and the sub-tropical convergence (STC), and then migration north along the western Australian coast to Indonesia^25^ (Fig. 1). There is also information on the dive behaviour of one whale during its migration north from the Perth Canyon area^26^. Four tags deployed at the Bonney Upwelling region and one deployed at Geographe Bay have also shown movements from these areas to the STC^27^. These studies provided evidence of links between Australia, STC and Indonesia, the latter likely representing the calving ground location for these pygmy blue whales^25^. In addition, genetic studies have shown evidence that blue whales utilising Perth Canyon, Geographe Bay and Bonney Upwelling belong to one breeding stock of the pygmy blue whale subspecies^12,13^.

There is very little information about the fine-scale movements and occupancy patterns of blue whales in the Great Southern Australian Coastal Upwelling System (GSACUS) (Fig. 1). This annually, spatially and temporarily variable, wide-ranging system develops over the austral summer in response to south-eastern coastal winds over Australia’s southern continental shelves and represents one of the largest seasonal coastal upwelling systems on the planet^28,29^. The GSACUS is represented by the Bonney Upwelling, which is its strongest upwelling centre, and other smaller upwellings that form west of the lower Eyre Peninsula and over the western Tasmanian shelf^28^ (Fig. 1). There is also uncertainty around the migratory route and destination for whales feeding in this region as the four previous tags deployed on blue whales in the Bonney Upwelling transmitted for a short period of time^27^. A potential migratory route to presumed breeding grounds is along the WA coast to Indonesia, as observed for blue whales tagged at the Perth Canyon^25^ (Fig. 1). Alternatively, or additionally, blue whales at the GSACUS may migrate along the east coast of Australia^23^ to wintering grounds in the north-west South Pacific Ocean^30^. This is perhaps unlikely as the unique song of pygmy blue whales feeding in New Zealand predominates in the western South Pacific^14,15^, suggesting that blue whales from this population may migrate up the east coast of Australia.

The aims of this study were to investigate the movements, occupancy patterns, and infer the behaviour of pygmy blue whales in the GSACUS, and after they leave the GSACUS, and identify potential environmental drivers of these behaviours. Altogether, we seek to inform the conservation management of these endangered whales by investigating spatio-temporal patterns and their drivers. This is particularly relevant in the GSACUS as the region includes main shipping routes, sustains major fisheries, and is heavily targeted for oil and gas exploration^31,32^—all of which are activities known to impact upon whales.

## Results

### Satellite tags and photo-identification

The vessel effort equated to 4236 km (tagging and support vessels combined) for 21 days that weather permitted working on the water out of 40 days at the study site. Five MK10 SPLASH and eight SPOT tags were deployed (Table 1). All 13 whales were adults, based on visual size, and one of them (#131139) had an accompanying calf.

**Table 1:** Details of satellite transmissions from 13 pygmy blue whales (*Balaenoptera musculus brevicauda*) tagged in the Bonney Upwelling region, Great Southern Australian Coastal Upwelling System, southern Australia, between January and March 2015, with genetically resolved sex, estimated pygmy ancestry, and information on previous photo-identification and biopsy.

Tag ID	Tag type	Tagging date	Track duration (days)	Number of 3-h SSM-estimated locations	Distance tracked (km)	Mean speed (km/h)	# Days at GSACUS (minimum)	Photo-ID SHBWC alias	Biopsy-ID	Sex	Estimated pygmy ancestry	Previous Biopsy/Photo-ID date	Previous Biopsy/Photo-ID location
131124	MK10	21/01/15	3	17	203	4.2	3	FLBW001	BU1-15	F	0.998	15/01/12	38.5341°S/144.7615°E
131125	MK10	22/01/15	82	452	2865	3.4	58	FLBW003	BU2-15	M	0.996	–	–
131123	MK10	22/01/15	93	741	5239	3.2	93	no photo-ID	BU3-15	M	0.997	25/03/09	38.4844°S/141.5939°E
131122	MK10	27/02/15	38	268	1866	3	35	FLBW007	no biopsy	U	NA	–	–
131139	MK10	02/03/15	53	418	3054	2.8	53	FLBW010	BU4-15	F	0.981	–	–
123227	Spot	02/03/15	62	328	1455	1.9	46	FLBW012	no biopsy	U	NA	–	–
123233	Spot	03/03/15	382	2021	9360	3.4	166	FLBW016	BU5-15	M	0.997	–	–
123235	Spot	07/03/15	61	409	3654	3.8	55	FLBW017	BU6-15	M	0.997	–	–
123234	Spot	10/03/15	140	687	4360	3.3	91	FLBW022	BU7-15	M	0.993	-	–
131177	Spot	10/03/15	243	545	3108	3	1	FLBW024	BU8-15	M	0.995	–	–
123230	Spot	13/03/15	25	92	510	3.3	14	FLBW020	BU9-15	F	0.998	25/03/10	37.6020°S/139.7642°E
123229	Spot	13/03/15	271	1492	15120	3.2	51	FLBW028	BU10-15	F	0.988	–	–
131174	Spot	13/03/15	49	389	1940	2.8	49	FLBW015	no biopsy	U	NA	–	–

All blue whales were satellite tagged in the Bonney Upwelling region between 139.6°E and 140.9°E (Fig. S1). Of the 13 tagged whales, 12 and 10 were successfully photo-identified and biopsy sampled, respectively. From 59 high quality photographs showing photo-identifiable individuals, an additional 17 blue whales (not tagged/biopsied) could be identified. Identification images of all the whales were uploaded to the Southern Hemisphere Blue Whale Catalogue (SHBWC) with aliases FLBW1–FLBW29.

### Subspecies and individual identification, and sex of tagged whales

All tagged individuals that were biopsy sampled had genetically estimated ancestry to the pygmy subspecies ranging from 98.1% to 99.8% (Table 1, Fig. S2). Based on simulations^33^, this indicates that all tagged individuals are ‘pure’ pygmy blue whales (Fig. S2). Sexing analyses showed there were four female and six male individuals (Table 1). Of the 10 biopsied whales, three (#131124, #131123, #123230) had been biopsied sampled previously in the Bonney Upwelling region and adjacencies by Flinders University researchers (Table 1). Excluding intra-season resamples, this was out of a total of 48 biopsy samples collected between 1995 and 2015 in the region by the researchers for the current and previous^33,34^ studies. There were no mismatches in the genotypes of resampled individuals, and they were genetically identified as of the same sex. Two of the three tagged whales that were resampled also had matching photo-identifications. For the third whale (#131123), photo-identification was not obtained during tagging.

### Movements and occupancy patterns

The tags transmitted between 21 January 2015 and 20 March 2016 (Table 1). Whales were tracked from three to 382 days (mean = 116 days ± 114) (Table 1), including gaps, with the last tag transmitting on 20 March 2016 (Table S1). Total distances tracked per individual ranged from 203 km to 15,120 km (mean = 4056 km ± 4072), and average speed ranged from 1.94 to 4.24 km/h (mean = 3.18 km/h ± 0.55) (Table 1).

**Figure 2:**
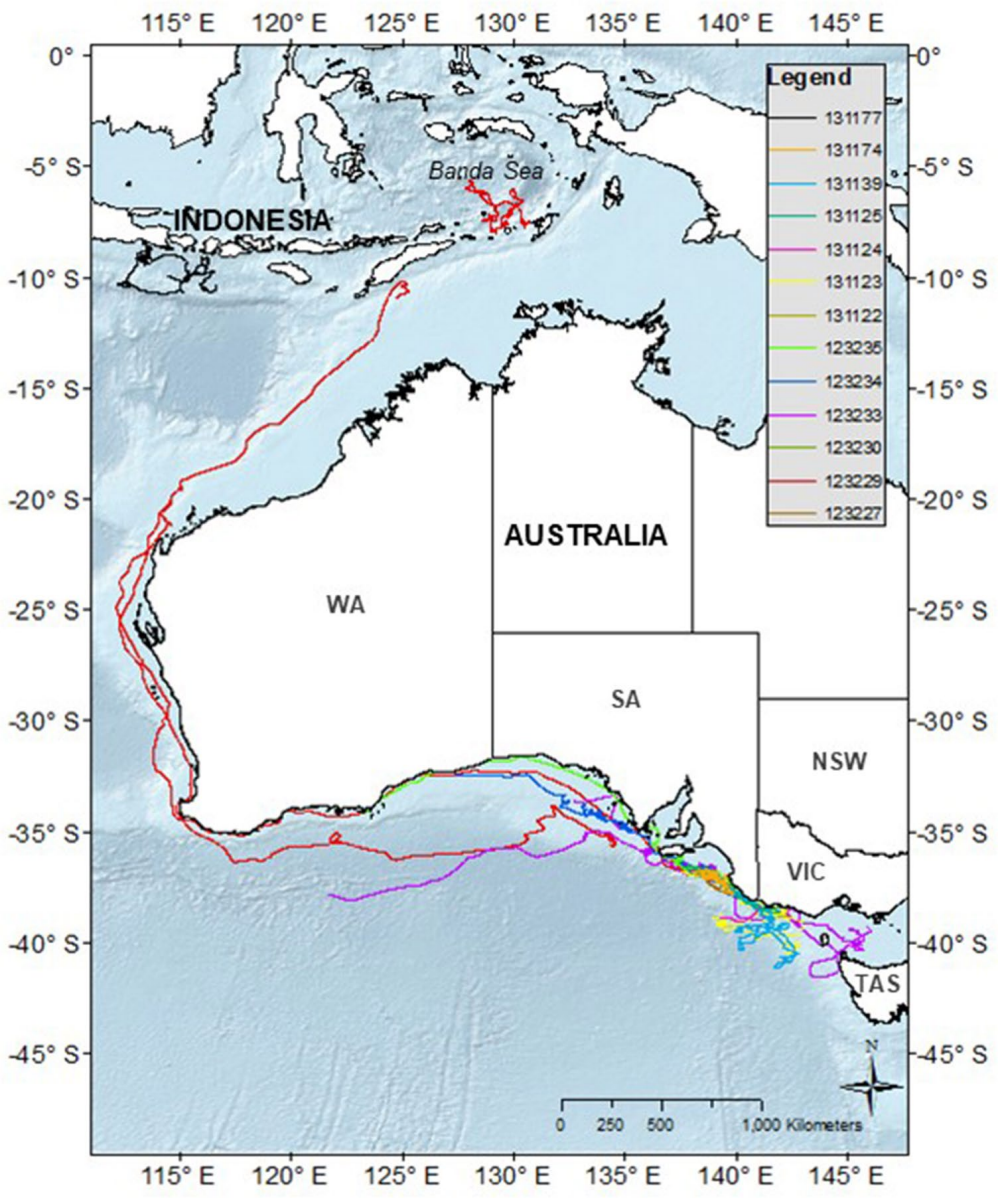
Individual hierarchical switching state-space model (hSSSM) derived locations of 13 pygmy blue whales (*Balaenoptera musculus brevicauda*) satellite tagged in the Bonney Upwelling region, Great Southern Australian Coastal Upwelling System, southern Australia, with whale tracks colour-coded by individual. *WA* Western Australia; *SA* South Australia; *VIC* Victoria, *NSW* New South Wales, *TAS* Tasmania. Map created in ArcGIS v.10 (available at https://www.esri.com/).

**Figure 3:**
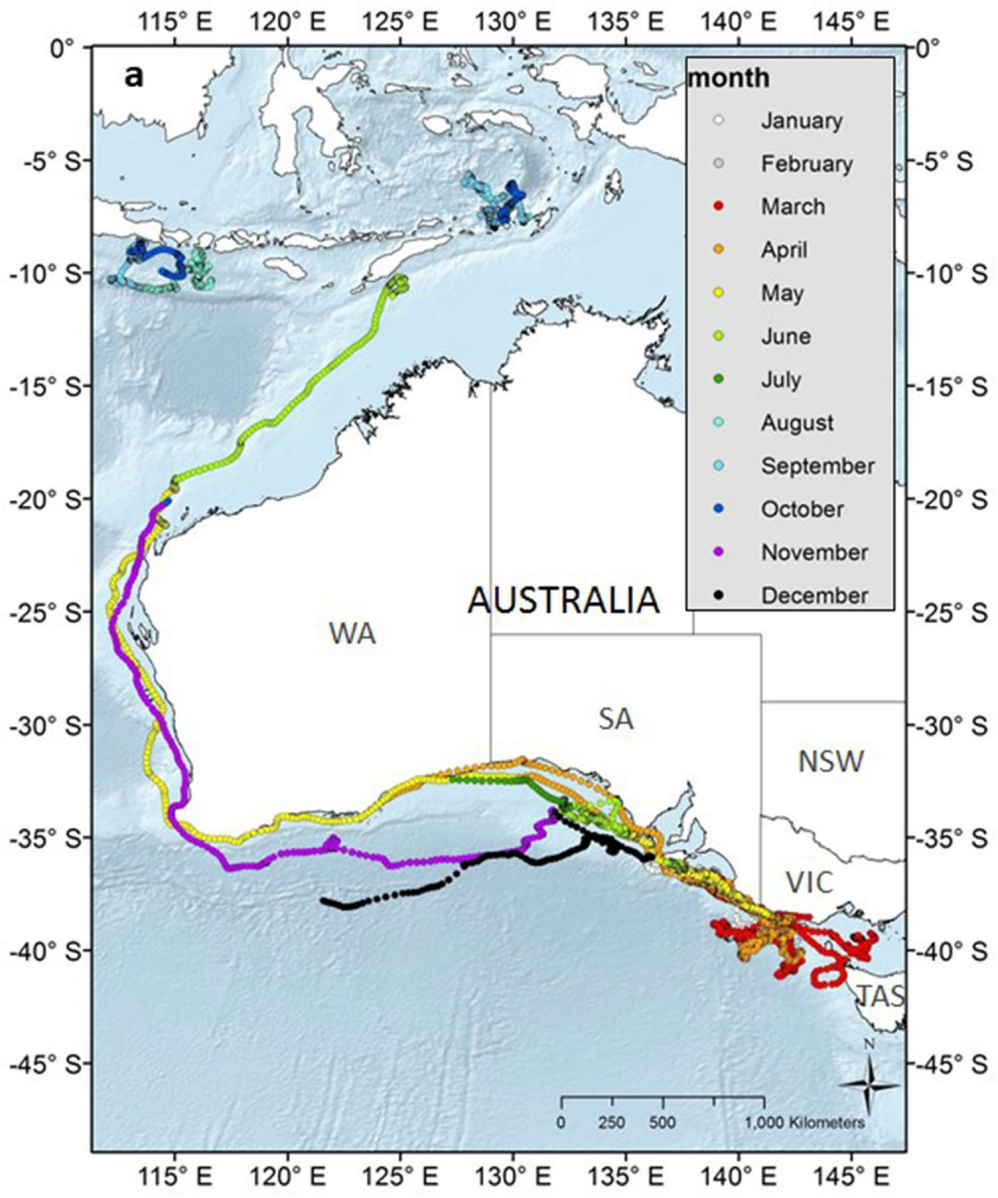

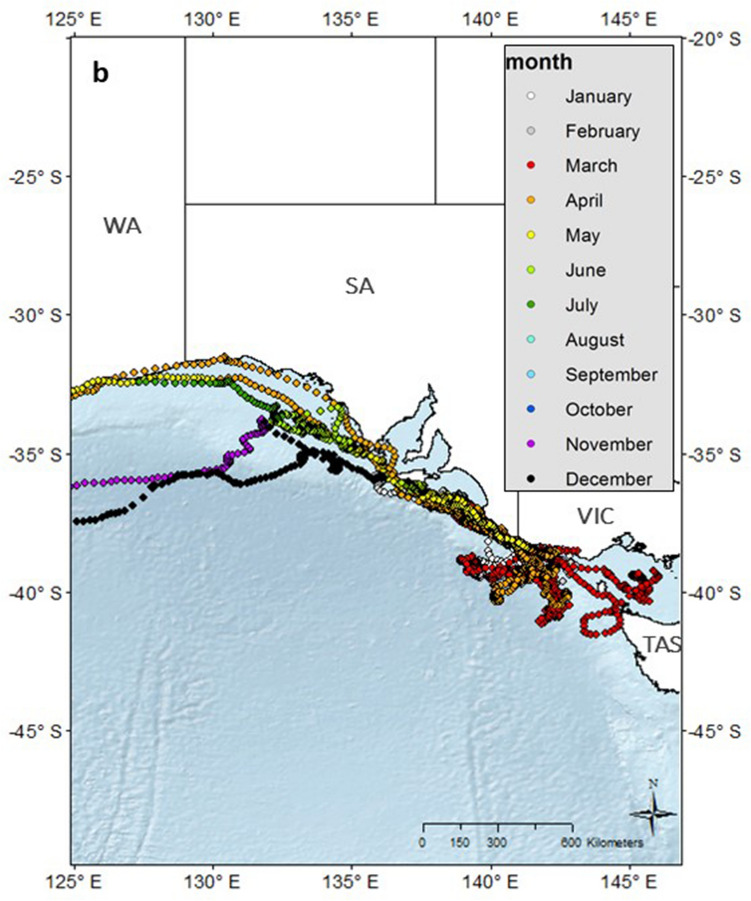
(**a**) Individual hSSSM derived locations of 13 pygmy blue whales (*Balaenoptera musculus brevicauda*) satellite tagged in the Bonney Upwelling region, Great Southern Australian Coastal Upwelling System GSACUS), southern Australia, with locations colour-coded by month. (**b**) Close-up of locations at the GSACUS (east of 125°E). *WA* Western Australia; *SA* South Australia; *VIC* Victoria, *NSW* New South Wales, *TAS* Tasmania. Maps created in ArcGIS v.10 (available at https://www.esri.com/).

**Figure 4:**
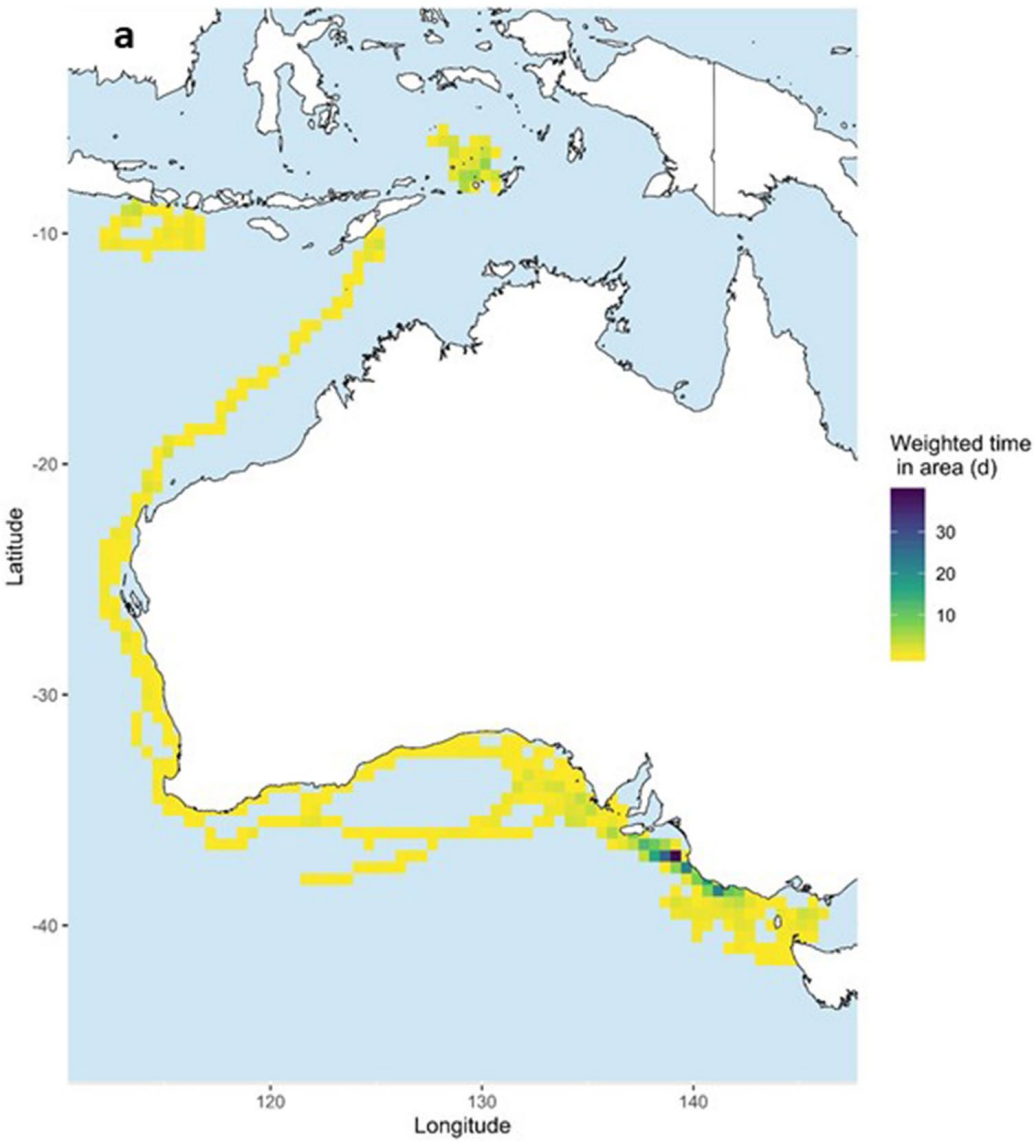

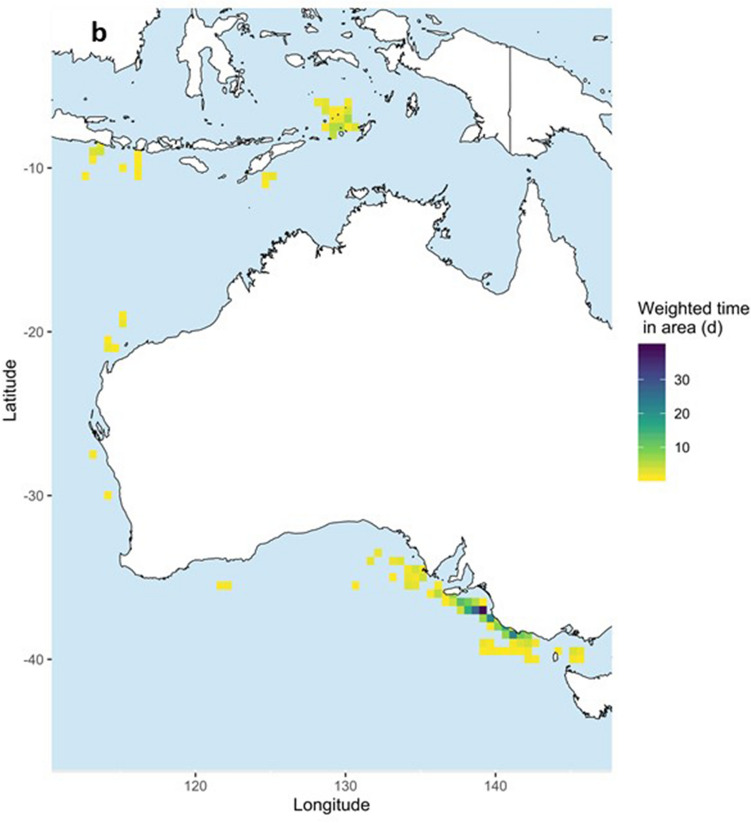
Occupancy patterns of 13 pygmy blue whales (*Balaenoptera musculus brevicauda*) satellite tagged in the Bonney Upwelling region, Great Southern Australian Coastal Upwelling System, southern Australia. Occupancy is calculated for each whale as weighted days spent in 0.5 × 0.5 degrees cells based on the individual hSSSM derived locations. (**a**) All weighted days and (**b**) restricted to cells with ≥ 1 weighted day. Maps created in R v.3, raster and rasterVis R packages ( available at https://www.r-project.org/).

After tagging, whales remained in the Bonney Upwelling and adjacent areas in the GSACUS (east of 125°E) from at least January to July 2015 (Figs. 2, 3a, Figs. S3–S15; Table S1), and between at least three to 107 days (mean = 54.58 ± 29.4), excluding a tag that failed to transmit until August 2015 (Tables 1, S1). In November and December 2015, respectively, two tagged whales (#123229, #123233) were recorded returning to the GSACUS, remaining there until at least December 2015 and March 2016 (Figs. 2, 3, Figs. S3 and S4), and spending at least 69 and 166 days in the region between the two annual feeding seasons (Table 1, Fig. S3). In general, the whales’ movements in the GSACUS ranged mostly from eastern SA, over the continental shelf south of Kangaroo Island, to between mainland Australia and Tasmania (TAS), with a few whales (#131123, #131124, #131139) performing some movements to the continental slope and the deep-sea (Figs. 2, 3, Figs. S8, S10, and S11). Considering individual deployment durations, the continental shelf of the Bonney Upwelling region showed the highest occupancy rate by the whales (Fig. 4).

Four tagged whales were observed leaving the Bonney Upwelling region in 2015 (#123229, #123233, #123234, #123235) (Figs. S3, S4, S5 and S13). They did so between late April and July, moving in a westward direction while off the southern Australian coast (Figs. 2, 3a). One of these whales (#123229) then moved north along the western Australian coastline (Figs. 2, 3a, Fig. S3). This whale reached waters south of West Timor by mid-June, then transmitted again in the Banda Sea where it remained from August to at least October 2015 (Figs. 2, 3a, Fig. S3). One tag that failed to transmit soon after tagging (#131177), started transmitting when the whale was in waters south of the Indonesian islands of Java, Bali and Lombok, remaining in the region from at least August to October 2015 (Figs. 2, 3, Fig. S6). Waters around Indonesia, in the Banda Sea and south of Java, Bali and Lombok, showed the second highest occupancy rates by the whales (Fig. 4).

One tagged whale (#123229) partially transmitted on its southbound migration between November and December 2015, following a similar migratory path south along the western Australian coast (Figs. 2, 3a, Fig. S3). This whale was particularly close to shore around the south-west corner of Australia, then went offshore staying north of the subtropical convergence (STC), and finally moved to the edge of the continental shelf in southern Australia where the tag stopped transmitting (Figs. 2, Fig. S3). Also, one whale that stopped transmitting soon after leaving the Bonney Upwelling region (#123233) re-started transmitting in December 2015, when it was south of southern WA (around the northern boundary of the STC) (Figs. 2, 3a, Fig. S4). It then moved to the continental shelf and returned to the Bonney Upwelling region from January to at least March 2016 (when the tag stopped transmitting), utilising a similar area to which it had utilised the previous year (Figs. 2, 3, Fig. S4).

### State-space model and behavioural states

**Figure 5:**
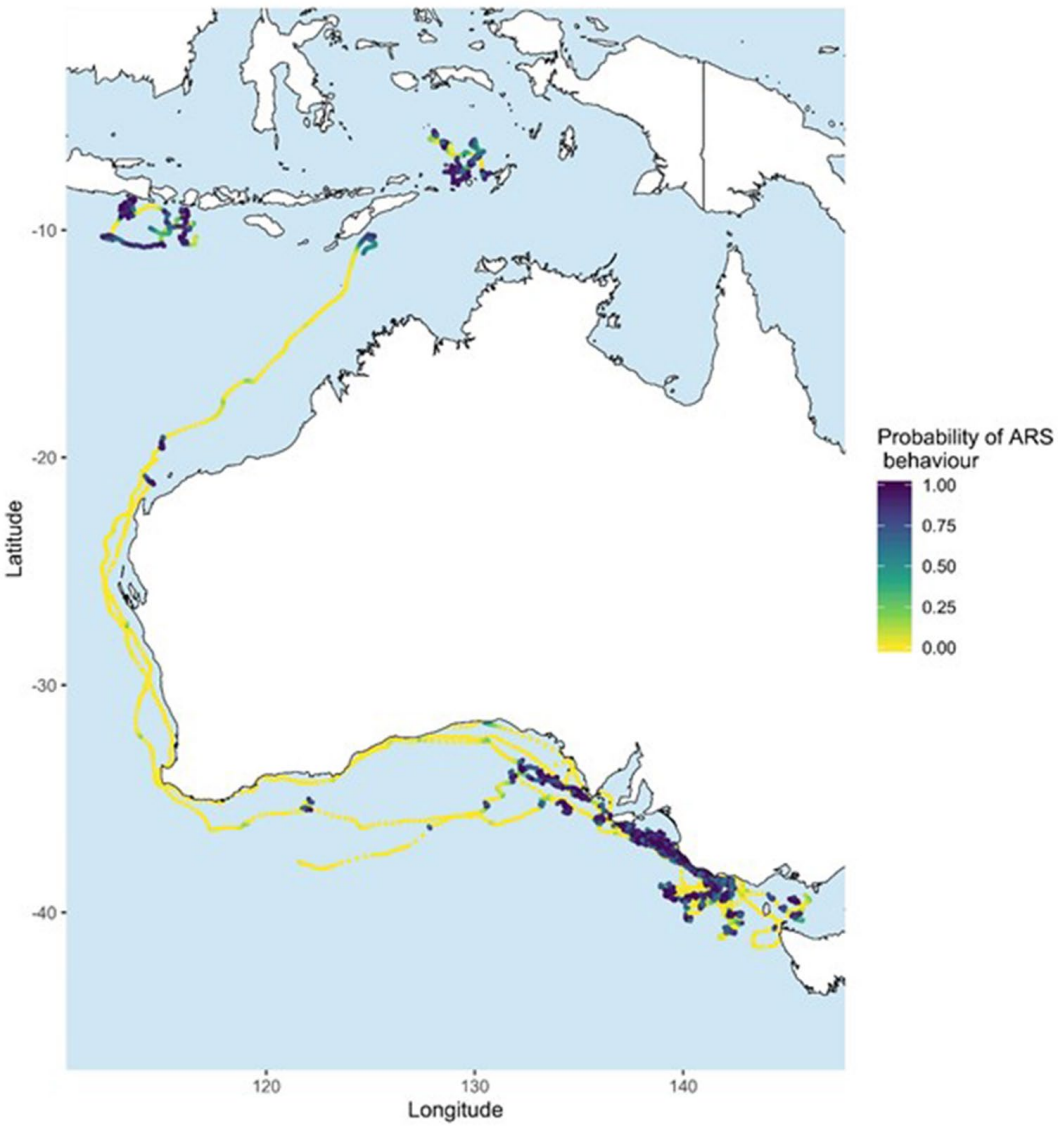
hSSSM derived locations and estimated probability of ARS behaviour of 13 pygmy blue whales (*Balaenoptera musculus brevicauda*) satellite tagged in the Bonney Upwelling region, Great Southern Australian Coastal Upwelling System, southern Australia. Values close to 1 indicates a high probability of the whale being in ARS behaviour. Map created in R v.3, raster and rasterVis R packages (available at https://www.r-project.org/).

The probability of area restricted search (ARS) behaviour was greater in the GSACUS region, Indonesia (Banda Sea, and south of Java, Bali and Lombok) and West Timor (Fig. 5) as shown by a hierarchical switching state-space model fitted to the satellite tagging data. Other areas where ARS behaviour probability was high included two smaller areas in WA, north of Northwest Cape, and waters south of West Timor. By contrast, the probability of ARS greatly decreased after whales left the GSACUS region, along southern WA and western WA, and on return to southern Australia (Fig. 5).

### Seascape correlates of inferred ARS behaviour in the GSACUS

**Figure 6:**
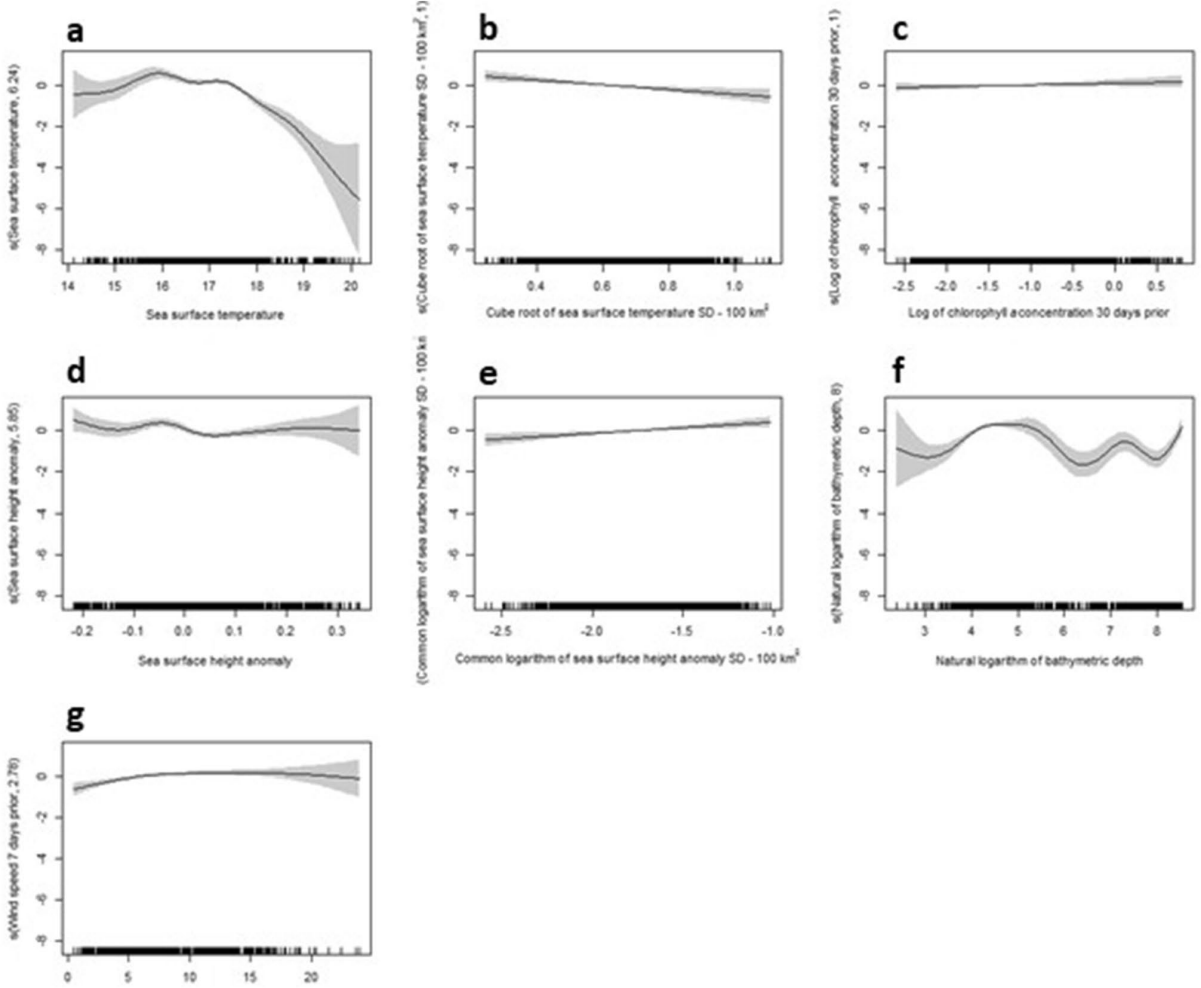
GAMM smooth terms of the environmental predictors of pygmy blue whale (*Balaenoptera musculus brevicauda*) ARS behaviour in the Great Southern Australian Coastal Upwelling System (east of 125°E) selected in the top model. X-axes show location of observations, while Y-axes show effects of smooth functions on behaviour probability. Solid lines represent the smooth estimates, while areas within dashed lines represent estimated smooth standard errors. (**a**) sea surface temperature (sst), (**b**) sea surface standard deviation (sdsst), (**c**) log of chlorophyll *a* average (30-day prior), (**d**) sea surface height anomaly (ssha), (**e**) log10 of sea surface height anomaly standard deviation (sdssha), (**f**) log of depth, and (**g**) wind speed (7-day prior).

The inferred behavioural state of whales in the GSACUS region (transit, *b* = 1, n = 1038; ARS, *b* = 2, n = 3907) was significantly associated with several seascape variables as indicated by generalised additive mixed effects models (GAMM) (Table 2). The final top GAMM (coefficient estimate = 1.5713 ± 0.3301 S.E., z-value = 4.76, P = 0; R^2^ adjusted = 0.153) included the variables sea surface temperature (SST), sea surface temperature variability (SST_SD), depth, wind speed (7-day prior at nominated point location (Nieblas et al. 2009)), sea surface height anomaly (SSHa), sea surface height anomaly variability (SSHa_SD), and chlorophyll *a* (30-day lag) (Table 2). ARS behaviour was more commonly observed at 14 °C to 17 °C SST, and in areas with lower SST_SD, deeper bathymetry over the shelf, prevailing wind speeds from 12 to 18 km/h, lower SSHa but higher SSHa_SD, and higher chlorophyll *a* concentration 30 days prior (Fig. 6). The most resilient seascape variables to the spatial uncertainty were SST, SST_SD, depth and the 7-day prior wind speed, followed closely by the SSHa and SSHa_SD, and to a much lesser extent the 30-day prior chlorophyll *a* concentration (Table 2).

**Table 2:** Environmental predictors of pygmy blue whale (*Balaenoptera musculus brevicauda*) ARS behaviour in the Great Southern Australian Coastal Upwelling System selected in the top GAMM.

Variable	Chi-square	p-value	Number of times p < 0.05
SST*	155.717	0	100
SST_SD*	26.729	0	100
Depth	157.219	0	100
wind speed**	54.468	0	99
SSHa*	54.802	0	91
SSHA_SD*	38.616	0	94
Chlorophyll a***	38.616	< 0.001	38

Shown is the approximate significance of smooth terms based on Chi-square statistics, and the number of times each variable was significant (p < 0.05) using 100 random subsets of the bsam (hSSSM) posterior distribution. This determines the variable’s resilience to spatial uncertainty.

## Discussion

Our study provides novel as well as corroborative information about the movements, occupancy patterns, and behaviour of pygmy blue whales in the eastern Indian Ocean. This includes their migratory routes from southern Australian foraging grounds, to a Western Australian migratory corridor, and to presumed breeding grounds in waters around Indonesia. Combined with previous studies, our results assist in defining the spatio-temporal distribution of this population of pygmy blue whales, and in identifying areas of high occupancy and biological importance off Australia and Indonesia, and perhaps West Timor. This extends information about pygmy blue whale BIAs available within the Australian National Conservation Values Atlas. Our study also reveals seascape correlates of ARS behaviour for pygmy blue whales while in the GSACUS foraging grounds. Together with additional movement data, this could be used to predict future whale presence and behaviour given forecasted effects of climate change, e.g.^35^, including to coastal upwelling systems^36^. Importantly, the combined movement ecology data can assist in mitigating potential impacts from human activities on this recovering pygmy blue whale population.

### Foraging grounds in the Great Southern Australian coastal upwelling system

In the GSACUS, most tagged whales remained over the continental shelf, utilising this region from at least January to July. This was the area of highest occupancy by the whales, with one whale returning to the Bonney Upwelling in January the year after and remaining there for at least three months. This timing coincides with the upwelling season, which generally occurs from November to March each year^37^. In the GSACUS, whales predominantly carried out ARS behaviour**,** including in secondary upwelling areas such as around Kangaroo Island off South Australia, which support spatially smaller upwelling centres^38^. These areas may be important to the whales depending on the intensity of the upwelling in these locations in a given year, and the timing and intensity of the adjacent Bonney Upwelling, which follows periods of onset, sustained, quiescent and downwelling^37^. During fieldwork in the Bonney Upwelling region, whales were observed several times foraging on krill swarms at the surface (Attard, Bilgmann, Paton, pers. obs.), and were presumably also foraging in the other upwelling areas based on the estimated ARS behaviour. These movements and behaviour corroborate previous suggestions that blue whales aggregate to feed in the wider GSACUS, between the Great Australian Bight to Bass Strait, from late spring to autumn each year^23^. Sighting data from aerial surveys carried out in previous years suggested that between November and December blue whales utilise mainly slope waters in the western part of the GSACUS, south of Kangaroo Island and Eyre Peninsula, while from January to April they are usually found in shelf waters of the central and eastern parts, between Cape Jaffa and Cape Otway^23^ (Fig. 1).

Seascape correlates of ARS behaviour for these whales suggested the importance of sea surface temperature, sea surface height anomaly, wind speed and chlorophyll *a* concentration as proxies of upwelling productivity and presence of krill patches. Although characterizing the environmental parameters that influence the search behaviour of marine predators remains challenging as it is usually not possible to directly measure prey distribution across the scale over which movement occurs, several other blue whale studies have found the importance of similar variables, and temperature ranges. A previous study in the GSACUS found that the presence of blue whales was mainly associated with sea surface temperature followed by chlorophyll *a* concentration, with other variables of lesser importance^23^. In our study, ARS behaviour in the GSACUS was more commonly observed in areas between 14 and 17 °C SST, a thermal range previously suggested to relate to pygmy and Northern blue whale (*B. m. musculus*) thermal preference and/or the presence of krill swarms^23,39,40^. The temperature range observed for pygmy blue whales ARS behaviour in the Bonney Upwelling is indeed within the suggested optimal temperature range of its prey in the region, *N. australis* (12–18 °C, Blackburn^41^). In the California Current Ecosystem (CCE), a region also characterised by seasonal upwelling, Northern blue whale (*B. m. musculus*) presence and ARS behaviour were associated with similar environmental variables to those in our study, although a different krill species is found there. This included sea surface temperature, chlorophyll *a*, sea surface height anomaly and bathymetry^39,42^. In the Azores region, North Atlantic, habitat niches of both Northern blue and fin whales were also strongly associated with primary productivity and similar sea surface temperatures^40^.

In the CCE, recent modelling studies suggest that blue whales exhibit strong foraging fidelity between years^42^. This means blue whales not only track contemporary resources, but may also use memory of long-term climatological conditions^6^. Movement data suggests that whales in the CCE mainly forage in areas with higher long-term chlorophyll *a* concentrations compared to contemporaneous measurements^6^. This could perhaps explain why chlorophyll *a* concentration, which was also a significant predictor in our model, was not as resilient to the spatial uncertainty as the other seascape variables. The Bonney Upwelling region is known to show variable chlorophyll *a* concentrations from year to year due to synoptic weather variability^28^, thus whales could be better off by moving to areas of known, repeated past productivity within the expansive GSACUS. Alternatively, a 30-day lag of chlorophyll *a* concentration may not be a reliable predictor of krill distribution due to the highly dynamic nature of the upwelling system. In our study, we were also able to capture the impact of wind forcing on the system’s dynamics and its effect on whale ARS behaviour. This was achieved by using a 7-day prior of wind speed at a point location suggested to represent the coastal wind that directly influences upwelling along the Bonney region^37^.

In the Bonney Upwelling, whales were observed to carry out ARS behaviour mostly over the continental shelf, an area where their prey *N. australis* is known to aggregate and where whale sightings concentrate^23^. However, a few whales, including the female accompanied by a calf, were observed to utilise areas over the slope and deep-sea. This suggest that whales may also be feeding in mesopelagic waters and potentially on other prey species. In an aerial study^23^, blue whales in the Bonney Upwelling were mainly observed over the continental shelf, but sightings further west in the GSACUS, off Eyre Peninsula, were generally over the slope. The latter pattern is similar to the distribution of several rorqual species, including Northern blue whales, in the north-western Iberian Peninsula where they concentrate at the edge of the continental slope^43^. The eastern part of the GSACUS is characterised by a high abundance of krill, with dominance of *N. australis* over the shelf, *N. australis* and *Euphasia similis* over the slope, and *Nematoscelis megalops* in the oceanic environment^31^. This suggests that pygmy blue whales may be feeding on these other krill species whilst in deeper waters.

### Migration from southern Australia to wintering grounds off Indonesia, and potential feeding en route

A low probability of ARS behaviour (i.e. high probability of transiting behaviour) was mainly observed between April and June, and then between November and December, suggesting that the pygmy blue whales were mainly migrating during those times. The migratory behaviour after leaving the Bonney Upwelling and the GSACUS was initially in a westward direction along southern Australia, and then northwards along Western Australia, before reaching waters of West Timor and Indonesia. This provides evidence on the migratory route of whales from the Bonney Upwelling and GSACUS foraging grounds, and further evidence of the breeding ground locations, the latter representing the area of second highest occupancy by the whales. The northbound migratory route observed for whale #123229 was like the path observed for pygmy blue whales previously tagged in the Perth Canyon^25^. This suggests that whales from the GSACUS and Perth Canyon are likely to use the same breeding ground locations around Indonesia (Banda and Molluca Seas, and possibly south of the Islands of Java, Bali and Lombok), and corroborates genetic data showing that the whales belong to the same genetic population^12,34^.

While whales mostly displayed low probability of ARS after leaving the GSACUS, high probability (> 0.95) of this behaviour for whale #123229 was estimated in a few other areas, in addition to Indonesian waters: two areas north of Northwest Cape in Western Australia, and one south of West Timor. These could represent stopover areas where whales may spend some time feeding while *en route* to breeding grounds. Further research around Timor, as well as south of Java, Bali and Lombok, where a first recording was made for this population (this study), would be important to clarify the regions’ significance for blue whales and whether they may represent unknow feeding/breeding areas. Feeding *en route* to breeding grounds is similar to what has been proposed based on tracks and dive data from pygmy blue whales satellite tagged in the Perth Canyon feeding ground^25,26^, and may occur if they migrate through areas of high krill concentrations.

The oceanographic conditions on the western Australian coast indicate that blue whales could indeed forage in several places during their northern migration to Indonesia, depending on the environmental conditions each year. The Western Australian coast is dominated by the Leeuwin Current system, which is characterised by the near surface, southward flowing warm water Leeuwin Current, the deeper, northward flowing Leeuwin undercurrent, as well as ephemeral coastal counter currents^44,45^. In this region, various transient upwelling events occur over the continental shelf in spring and summer, particularly north of the Perth Canyon^46^. Several euphausiid species are present, including the abundant *Euphausia recurva* and *Stylocheiron carinatum*^47^, which may be prey for blue whales. Several recent studies have shown supplemental feeding by whales during migration^48,49^, suggesting that the traditional ‘feast and famine’ model for baleen whales that migrate between breeding and feeding grounds may not apply to pygmy blue whales and some other baleen whales.

The tagged whales from our study that travelled to Indonesia either travelled specifically to the Banda Sea region, as was the case for some of the whales tagged at the Perth Canyon^25^, or south of the Indonesian islands of Java, Bali and Lombok, for which no link to Australian pygmy blue whales had been previously made. In Indonesian waters, whales mostly showed a high probability of being in ARS behaviour. This is possibly associated with breeding, but potentially also foraging as the state-space models cannot currently differentiate between the two behaviours. All of these places represent highly productive upwelling areas during the southeast monsoon season between July and September each year^50–52^, and the upwellings are particularly strong and longer during El Nino-Southern Oscillation years, such as in the 2015 tagging year^53^. These months coincide with when pygmy blue whales are in the region^25^, and perhaps whales take advantage of these productive waters to also feed during the breeding season.

Insights about movements during the southbound migration, for which little information is currently available, was gathered through one of the whales with a longer tag transmission, representing the first comprehensive southbound track of a pygmy blue whale from this population. This whale (#123229) was observed moving south along the western Australian coast between November and December, following a similar path to its northbound migration. This corroborates acoustic data, which suggests that the southbound migration of pygmy blue whales along Western Australia occurs mostly far from the coast, but with some whales migrating closer to shore (i.e. up to about 400 km^54^). The same whale then moved closer to shore in Geographe Bay, where blue whales are commonly observed from shore travelling southward at this time of the year^8^, before moving around Cape Naturaliste and Cape Leeuwin.

The southbound whale (#123229), as well as another whale (#123233) whose transmission re-started in December, spent time offshore in southern Australia around the northern boundary of the STC during November and December 2015. This region has been suggested as a major foraging ground for these whales^8,23,27^. One whale tagged in Perth Canyon in 2011 was also observed to utilise the STC from December to likely February^25^, while another previously tagged in the Bonney Upwelling in 2005 utilised this area in at least April^27^. Additional tagging studies of this population would be beneficial to clarify variants to the southbound migration as well as movements west of WA into the Indian Ocean and STC, as suggested by several lines of evidence^8^. The two whales (#123229, #123233) then returned to the Bonney Upwelling from January to at least March of 2016. There they utilised a similar area to the previous year, suggesting between-year individual site fidelity to this foraging ground.

Together with other studies including visual surveys, past whaling catches, acoustics and genetics, this study provides further evidence of at least two pygmy blue whale populations utilising Australian waters. The pygmy blue whales from the GSACUS travelled west along the southern Australian coast to then migrate north to Indonesian waters. This is mostly the same route as pygmy blue whales tagged in the Perth Canyon in 2009 and 2011, albeit temporally distinct as whales were foraging in the Perth Canyon between March and April of those years^25^, while whales tagged in the Bonney Upwelling in 2015 were still in the GSACUS at this time, and at least one of these passed the same latitude as the Perth Canyon in May (Figs. 3a, Fig. S1). All these whales seem to be part of an eastern Indian Ocean population, which ranges from at least southern Australia, the STC, and Western Australia to West Timor and Indonesia^12,14,16,21–23,27,34^. The other pygmy blue whale population seems to represent a western Pacific Ocean population, which ranges from at least south of New Zealand, including around New Zealand where they feed, to the east coast of Australia where they perhaps migrate, and to at least Tonga where they perhaps breed^14–16,55^.

### Application of movement ecology data for conservation and management of Eastern Indian Ocean pygmy blue whales

This study provided novel movement ecology data for eastern Indian Ocean pygmy blue whales which forage in the GSACUS, southern Australia, and migrate through southern and western Australia, and West Timor, to presumed breeding grounds in Indonesia (Banda Sea and possibly south of Java, Bali and Lombok). These regions include a range of human activities that may impact upon whales and hamper their population recovery, such as major shipping lanes^56^, extensive fisheries^57^, oil and gas exploration^58^, and climate change^59^. This study thus informs about the spatio-temporal scale of BIAs for these blue whales, which can be used to examine potential exposure to human activities.

BIAs, which were designed to assist decision-making under Australia’s *Environmental Protection and Biodiversity Conservation Act 1999* (EPBC Act) and are found within the National Conservation Values Atlas of Australia, can now be extended for pygmy blue whales with the movement ecology data presented here. For example, pygmy blue whale distribution, foraging and migration areas in its presently recognised BIAs (Fig. 1; also accessible from the National Conservation Values Atlas of Australia^24^ under ‘Whale—Biologically Important Areas’, ‘Pygmy Blue Whale’) do not include many temperate waters south of Australia (near the STC), and tropical waters south of the Islands of Lombok, Bali and Java in Indonesia. Knowledge about BIAs are regularly used by the oil and gas industry and given the industry’s interest on exploration in the GSACUS, such as Bass Strait and Great Australian Bight, the information provided here is of high importance. For example, this assists industry with identifying the best time of the year for surveys and where whales are most likely to be found, such as for foraging in areas with SST of 14 °C–17 °C, and deeper bathymetry over the shelf in the Bonney Upwelling.

The movement data from this study also highlights the importance for these whales of the highly productive upwelling regions in southern Australia and Indonesia. To protect the whales and to facilitate population recovery, these areas could be targeted by these countries as part of the establishment and enhancement of their respective marine protected areas and networks as outlined in Australia’s *Marine Bioregional Planning* and Indonesia’s *Geographic Priorities for Marine Biodiversity for Marine Protected Area Development*.

Into the future, combining whale movement ecology data with matrices of risk exposure in dynamic environmental models could further assist with targeted spatio-temporal conservation management actions for these whales, e.g.^60^, but for this to be achievable additional movement data over several years would be required to improve model predictions.

Finally, the movement ecology information presented here is also relevant for the design of future blue whale population studies. These may need to consider potential biases associated with the wide spatio-temporal distribution of these whales, such as in studies applying mark-recapture or line-transect methods to estimate abundance and trends, e.g.^61,62^.

## Conclusions

This study confirms the importance of the GSACUS, including the Bonney Upwelling region, southern Australia, as a foraging ground for the eastern Indian Ocean pygmy blue whale population, and indicates the whales’ migratory route along southern and western Australia, and possibly West Timor, to their likely breeding grounds in the Banda Sea (and Molluca Sea), and perhaps south of Java, Bali and Lombok, in Indonesia. The results highlight many, but probably not all biologically important areas for this blue whale population, and further studies and movement data are warranted. However, this new information, together with other tracking, acoustic, sightings, genetics, and past catch data, substantially expand knowledge about the spatial and temporal distribution of this recovering blue whale population and its potential exposure to impacts from human activities throughout its range. All these data can contribute to various conservation management decision-making processes under the countries’ environmental legislations and plans, and for developing inter-governmental collaborations to protect these pygmy blue whales.

## Methods

### Fieldwork

Fieldwork was carried out in the Bonney Upwelling region, SA, between 7 January and 16 March 2015. Aerial surveys were initially used to locate aggregations of whales in the region and direct the research vessel to general locations where whales were present. Boat surveys were carried out for satellite tagging, photo-identification, and biopsy sampling in continental shelf waters up to 130 m deep and as far as 43 km from shore. For tagging, a 6.5 m rigid hull inflatable (RIB) vessel with two 70 hp outboard engines was used. As a support vessel for locating whales, and for work, health and safety purposes, an 8.5 m RIB with two 225 hp engines was used.

Fieldwork and procedures were carried out under research permits from PIRSA Ministerial Exemption S115, DEWNR research licence Q26268-1, Marine Parks permit MO00036-1, Commonwealth research permit 2013–0013, and animal ethics approval from Flinders University AWC approval E397. All experiments were performed in accordance with the relevant Australian guidelines and regulations.

#### Satellite tagging

A total of 13 satellite tags (Wildlife Computers, USA) were deployed on blue whales (Table 1; Fig. 1). These comprised five MK10 SPLASH tags that record geo-location, dive behaviour, dive depth and dive duration (dive data not included here), and eight light level SPOT tags that record geo-location, temperature, and light levels. Satellite tags were programmed without a duty cycle and with 45 s repetition intervals. The tags, sterilisation and deployment methods were those previously used and refined by the Australian Antarctic Division^25,48^. However, for these deployments, the anchor system was not articulated and the anchor—tag body interface was reinforced by a stainless-steel collar. The deployment involved a modified version of the Air Rocket Transmitter System (ARTS)^63^, with pressure set between 9 and 10 bars, and distance of approximately five meters from the whales.

#### Photo-identification

Photo-identification images of whales were taken from both the tagging and support vessels during fieldwork. Where possible, right and left dorsal sides of the whales were photographed, including the dorsal fin, for individual identification.

#### Biopsy sampling

Biopsy samples of tagged whales were collected immediately after a satellite tag was deployed. This was done using a Paxarms biopsy system^64^, with biopsy heads of size 5 mm × 10 mm. Samples were preserved in a 10% salt-saturated solution of DMSO.

### Data analysis

#### Argos data collection

Telemetry data were collected using the Argos satellite system. Argos data were downloaded once a week and collated over the time the tags were transmitting, with the presence of new data checked for up to one year after the last transmission. The Argos system apportions locations into 7 quality classes—3, 2, 1, 0, A, B, Z—loosely related to the number of successful tag transmissions received by a satellite during a pass^65^. All locations, regardless of quality class were retained for analysis via a state-space model (see below).

Since transmission from tags was intermittent for some of the whales, tracks were split into segments before analysis. This was done when gaps in transmission exceeded 48 h, and subsequent track segments with fewer than 20 observations were excluded from analysis. The splitting of tracks resulted in a total of 23 track segments for the 13 tagged whales (Table S1). Tag transmissions averaged once every 2.3 h across the 13 whales, and therefore a time step of three hours was chosen.

#### Behavioural state-space models and occupancy patterns

A hierarchical switching state-space model (hSSSM) was fit to the Argos-collected telemetry data using the bsam package^66^ in R^67^. The model estimated location states for each whale at regular 3-h time intervals, accounting for measurement error in the irregularly observed Argos surface locations and estimated the behavioural state (*b*) associated with each location.

The hSSSM enables joint estimation of parameters over multiple individuals, assuming individuals move according to a correlated random walk but with different diffusivity^66^. It uses a Bayesian approach to estimate the movement process parameters, i.e., move persistence (γ) and process variability (Σ), with the former representing the autocorrelation in speed and direction^68^. The model estimates two discrete behaviours: transiting (*b* = 1) and area‐restricted search (ARS; *b* = 2)^68^. Transit behaviour is usually characterised by faster, straighter movements, and is associated with short-distance travel or migratory behaviour. ARS behaviour is instead characterised by slower movements and more frequent changes in direction, which may be associated with foraging and/or breeding, depending on whether they are in foraging or breeding grounds^69,70^.

The hSSSM was fitted using Markov Chain Monte Carlo (MCMC) in JAGS^71^. Two chains were run to collect 30,000 samples each from the joint posterior distribution. This involved discarding the first 80,000 samples or each chain as burn-in and thinning the remaining samples by retaining every 30th sample to reduce autocorrelation. Posterior means of the location and behavioural states were calculated from a final set of 2000 MCMC samples. Convergence of the MCMC chains was assessed with trace plots of the chains to ensure adequate mixing, autocorrelation plots to ensure sufficient thinning had been used, and Gelman-Rubin shrink factor^72^ plots to judge whether the burn-in period was sufficiently long.

Maps displaying location of whale tracks and behavioural states as obtained from the analysis in bsam were created with ArcGIS, and the raster and rasterVis R packages, respectively. Since behavioural states are discrete (either 1 or 2), but their posterior means range continuously between 1 and 2, the behavioural state map was produced based on the probability of ARS behaviour (i.e., 1 subtracted from each posterior mean behavioural state estimate), with values close to 1 indicating a high probability of a whale being in ARS.

Occupancy pattern (time in area) was calculated for each whale as the number of days spent in each (0.5 × 0.5 degrees) cell, weighted by the whale's deployment duration (in days) divided by the longest deployment duration (in days), and then mapped with the raster and rasterVis R packages.

#### Generalised additive mixed effects models of behaviour-seascape associations

Generalised additive mixed effects models (GAMMs) were used to investigate potential associations between seascape variables and whales’ movement behaviour (response variable) in the GASCUS. The most probable discrete behavioural state was used (*b* = 1 or 2) and data were restricted to 30°S, 125°E (19 track segments from 12 whales, totalling 4945 locations) to represent the GASCUS, which is where most of the telemetry data was collected. The seascape predictor variables tested included sea surface temperature (SST), sea surface temperature standard deviation (SST_SD), sea surface height anomaly (SSHa), sea surface height anomaly standard deviation (SSHa_SD), chlorophyll *a* (Chla) average, eddy kinetic energy, bathymetric variables (depth, rugosity (depth standard deviation), slope (depth gradient), aspect (dominant direction of slope)), and wind variables (direction, speed at point location, and an upwelling index^37^) (details of data sources and resolutions can be found in Table S2). The seascape predictors were selected based on their potential to influence the search behaviour of blue whales as observed in previous studies, e.g.^39,42^, and included variables specific to the dynamics of the Bonney Upwelling^23,37^. The variables were trialled at different spatial (e.g. 50 vs 100 km^2^ SST and SST_SD) and temporal scales (e.g. 14-day vs 30-day chlorophyll *a* lag (based on 8-day averaged dataset), 7 and 14-day lags for wind direction and speed, 7 and 14-day prior for E and SE wind speed and upwelling index) (data not shown), before running the final models. Potential collinearity between variables was identified using variance inflation factors (VIF ≥ 3) and Pearson’s correlation coefficient (*ρ* ≥ 0.8). Collinearity was found between (i) SST_SD 50 and 100 km^2^, (ii) SSHa_SD 50 and 100 km^2^, (iii) Chla 8-day and 30-day, and (iv) Chla 14-day and 30-day lag (data not shown), with 100 km^2^ and 30-day scales kept for the modelling, and others excluded. The GAMM was fit using a binomial family with a logit link, and individual whales as a random effect, in R using gamm4^73^.

To account for uncertainty in both the behavioural state and the location estimates, we used a multiple imputation approach^74^. Following the approach described in Andrews-Goff et al.^48^, we re-sampled location and behavioural states from their joint posterior distribution. In brief, the first GAMM was fit with the summarised posterior mean for each location, and then 100 possible realisations were sampled from each of the two retained MCMC chains. With the new samples, the model was re-fit 100 times using newly extracted seascape variables at the new posterior sample locations. Significance of each seascape variable was then assessed by the number of times *p* < 0.05 was obtained out of the 100 iterations.

#### Photo-identification

All photo-identification images were screened for quality, sorted and whales were compared with each other. Right and/or left side images of individuals were then uploaded to the electronic SHBWC (at *bluewhalecatalogue.org*) using Flinders University Blue Whale (FLBW) aliases, along with information on geolocation, sex, and biopsy number, among others.

#### Genetic analysis

Genetic data were collected using a panel of 20 microsatellites from the tagged whales that were biopsy sampled. Tagged individuals that were previously sampled in our genetic dataset of 110 individuals from Australia (38 from whales sampled at the Bonney Upwelling region and 72 sampled at the Perth Canyon)^33,34^ were identified by identical multilocus genotypes or genotypes with up to two allele mismatches using EXCEL MICROSATELLITE TOOLKIT 3.1^75^. The sex of the individuals was determined by PCR amplification of a fragment of the genes ZFX/ZFY and SRY following the method of Gilston et al.^76^.

The subspecies identity of tagged individuals was confirmed to be pygmy blue whales using the microsatellite genotypes by following an assignment method as detailed in Attard et al.^33^. Specifically, the assignment method involved running the clustering method of STRUCTURE 2.3.4^77^ with the tagged individuals and, as a reference, 110 pygmy blue whales from Australia and 142 Antarctic blue whales from Antarctica (reference data from Attard et al.^33,34^) was chosen instead of other assignment methods (e.g. GENECLASS^78^) because it could also detect subspecies hybrids, which are known to occur in southern hemisphere blue whales^33^. STRUCTURE was run with the admixture model of ancestry, the correlated allele frequency model^79^, not using sampling locations as priors^80^, a burn-in of 100,000 iterations followed by 10^6^ iterations for each run, and ten independent runs of K = 2. Runs were summarised using CLUMPAK^81^ (main pipeline, default parameters) to obtain the estimated membership of each individual to the pygmy and Antarctic subspecies. STRUCTURE convergence was confirmed by the existence of only one mode of convergence when summarising runs using CLUMPAK. Tagged individuals were classified as 'pure' pygmy blue whales or 'pure' Antarctic blue whales if they had, respectively, at least 98% or no more than 12% of their estimated ancestry as originating from pygmy blue whales. This is because 99% of computer-simulated pure pygmy and pure Antarctic blue whales in Attard et al.^33^ had these values. Individuals with values in-between these were classified as having admixed ancestry.

## Supplementary information

Supplementary Figures.

Supplementary Table 1.

Supplementary Table 2.

Supplementary Legends.

## Data Availability

The satellite tagging metadata is available at the Australian Antarctic Data Centre, Data Management and Spatial Data Services. https://data.aad.gov.au/metadata/records/AAS_4101_pygmy_blue_whale_SSSM. The photo-identification data is available at Southern Hemisphere Blue Whale Catalogue (SHBWC) (at bluewhalecatalogue.org) under Flinders University Blue Whale (FLBW) aliases. The genetic data is available from the corresponding author upon request.
