# Supplementary material for: Movements and behaviour of blue whales satellite tagged in an Australian upwelling system
Source: Sci Rep. 2020 Dec 3;10:21165. doi: 10.1038/s41598-020-78143-2 (PMC7713308; doi:10.1038/s41598-020-78143-2)
Supplement: Supplementary file 1 — Supplementary Figures. [file 41598_2020_78143_MOESM1_ESM.pdf]

Movements and behaviour of blue whales satellite tagged in an Australian upwelling system

Luciana M. Möller, Catherine R.M. Attard, Kerstin Bilgmann, Virginia Andrews-Goff, Ian Jonsen, David Paton, Michael C. Double

Supplementary figures

Figure S1.

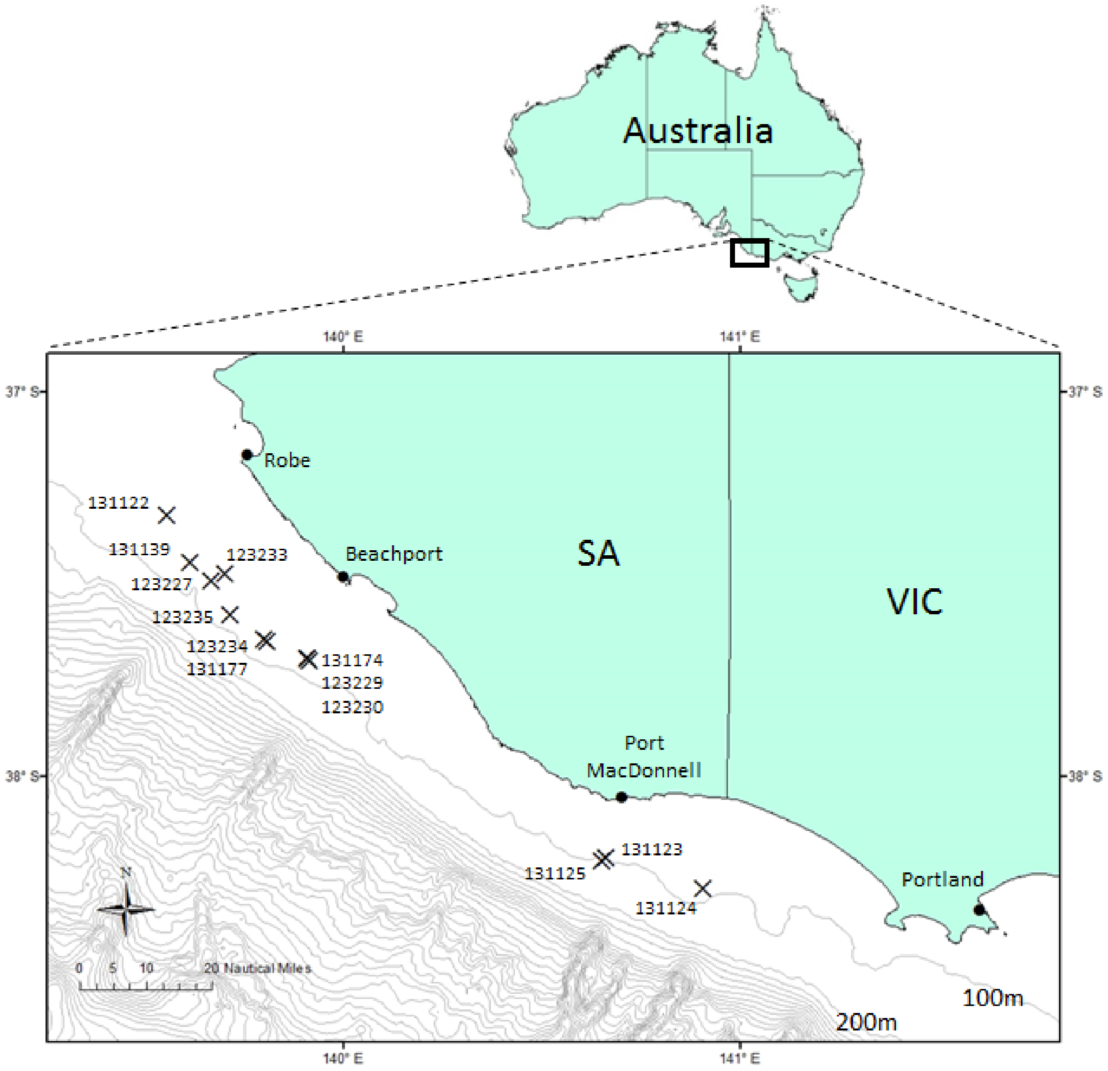

Figure S2.

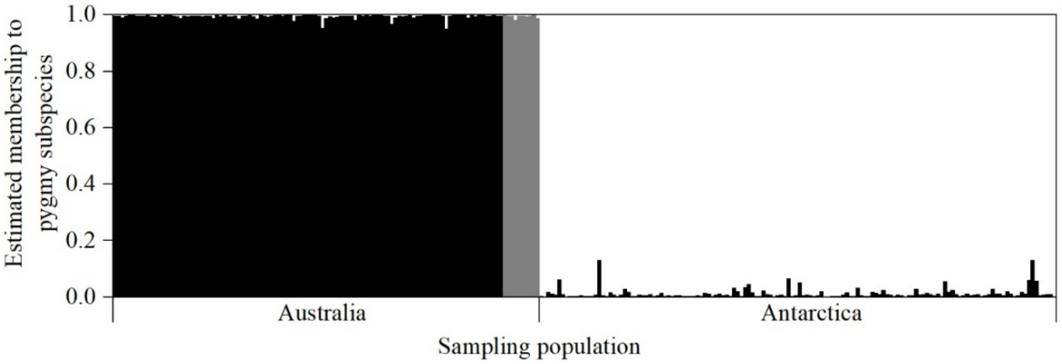

Estimated membership to pygmy subspecies
1.0
0.8
0.6
0.4
0.2
0.0
Australia
Antarctica
Sampling population

Figure S3.

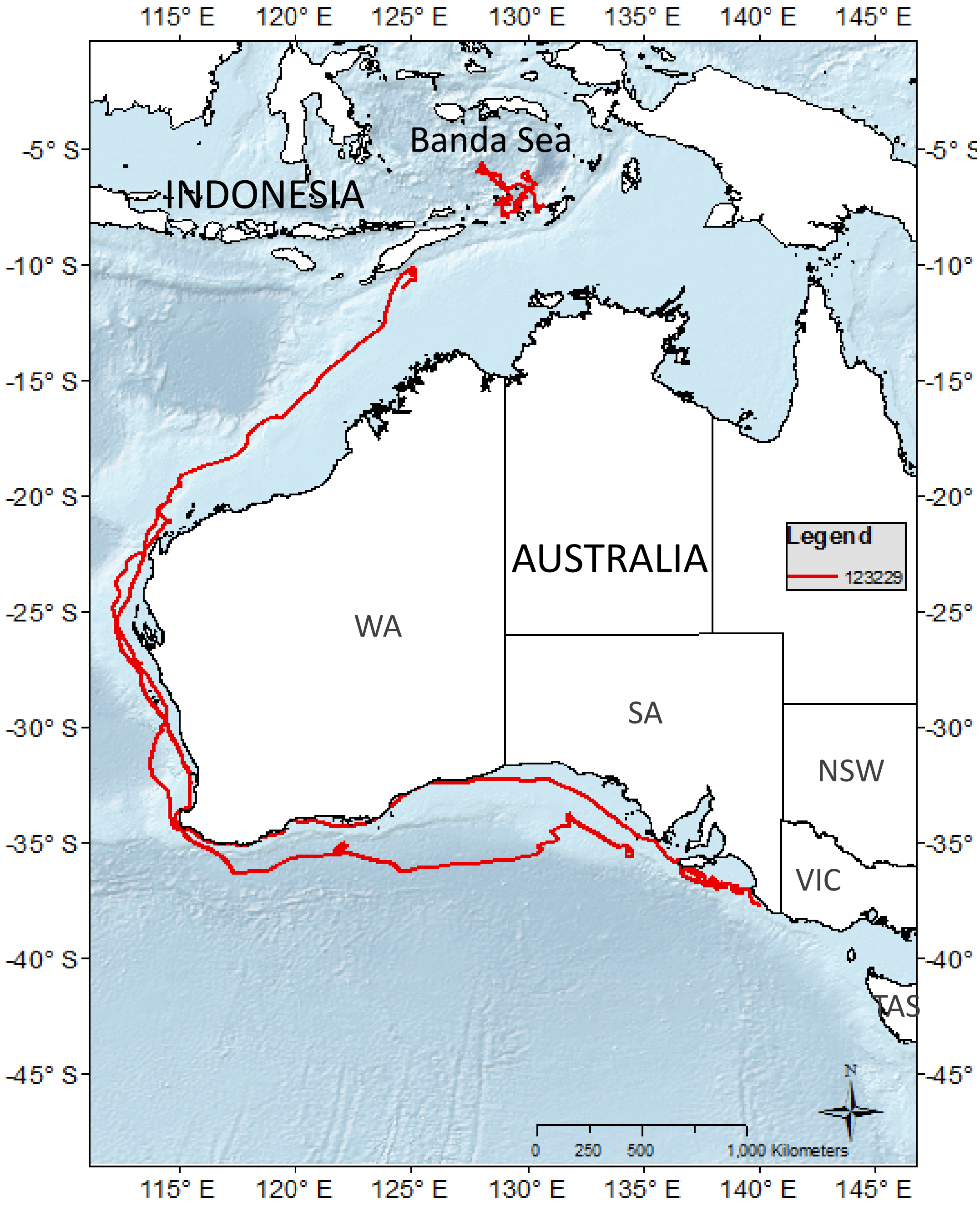
115° E
120° E
125° E
130° E
135° E
140° E
145° E
-5° S
-10° S
-15° S
-20° S
-25° S
-30° S
-35° S
-40° S
-45° S
Banda Sea
INDONESIA
AUSTRALIA
WA
SA
NSW
VIC
TAS
Legend
123229
0
250
500
1,000 Kilometers
N

Figure S4.

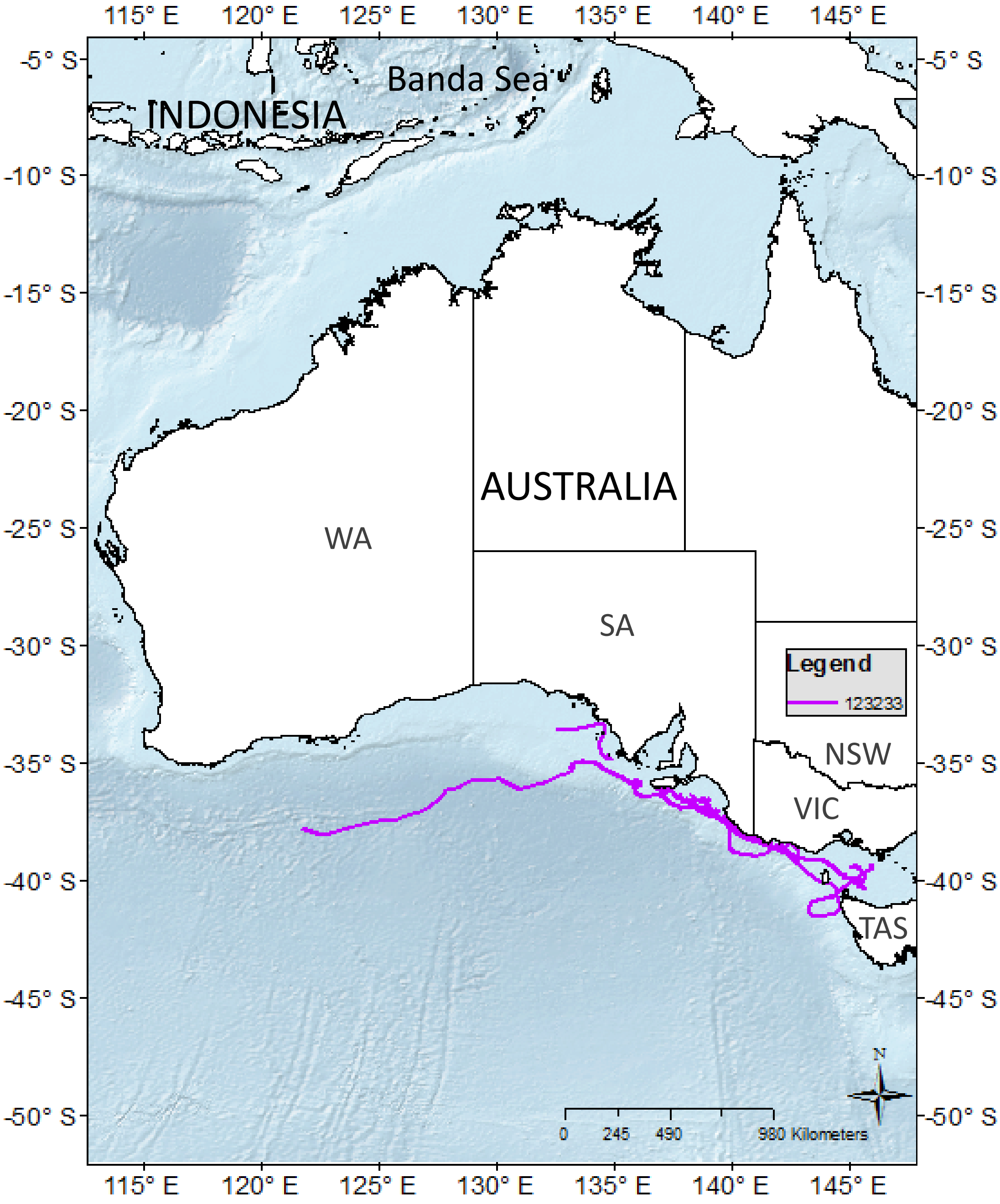
115° E
120° E
125° E
130° E
135° E
140° E
145° E
-5° S
-10° S
-15° S
-20° S
-25° S
-30° S
-35° S
-40° S
-45° S
-50° S
Banda Sea
INDONESIA
AUSTRALIA
WA
SA
NSW
VIC
TAS
Legend
123233
N
0
245
490
980 Kilometers

Figure S5.

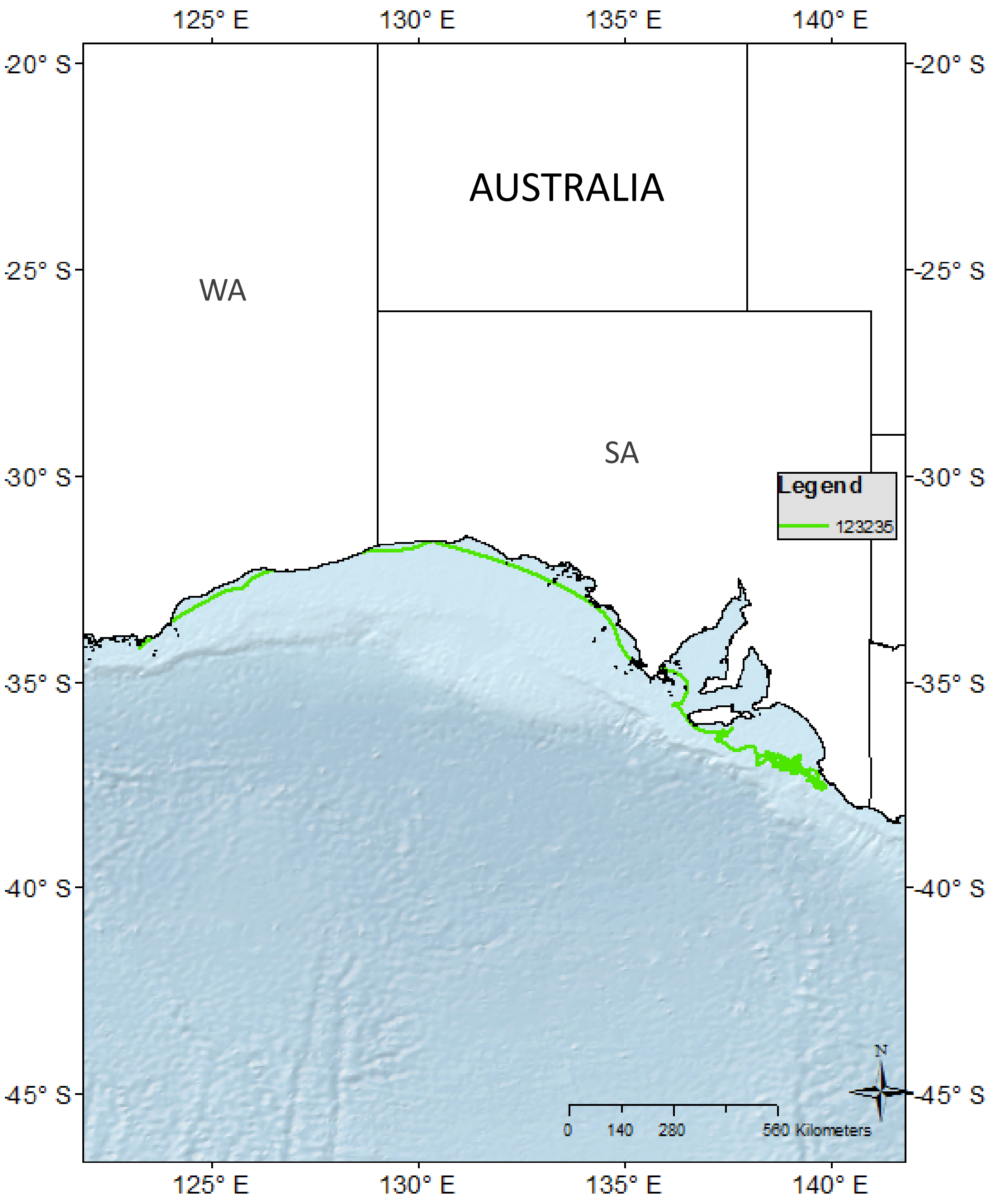
125° E
130° E
135° E
140° E
-20° S
-25° S
-30° S
-35° S
-40° S
-45° S
AUSTRALIA
WA
SA
Legend
123235
N
0
140
280
560 Kilometers

Figure S6.

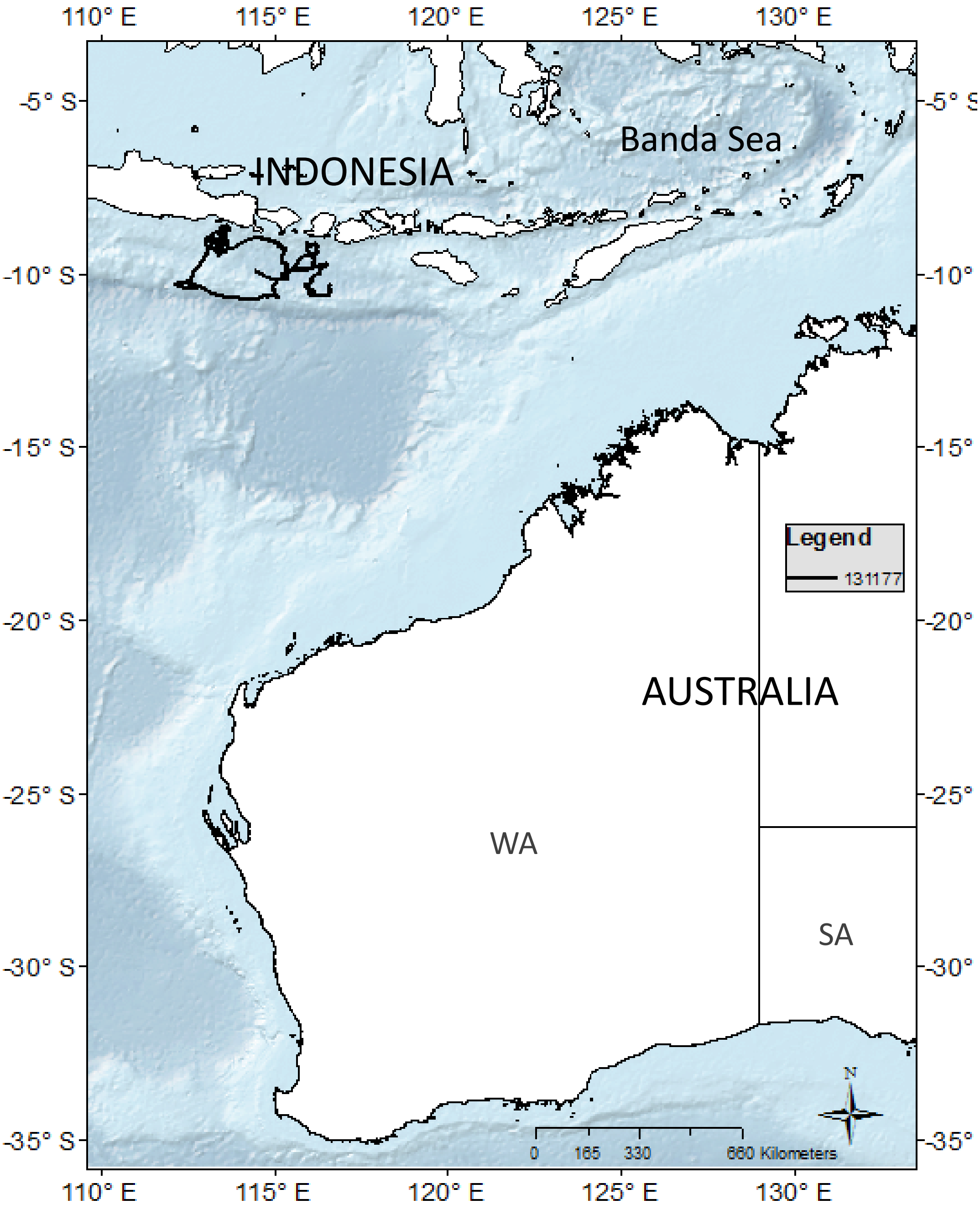
110° E
115° E
120° E
125° E
130° E
-5° S
-10° S
-15° S
-20° S
-25° S
-30° S
-35° S
INDONESIA
Banda Sea
AUSTRALIA
WA
SA
Legend
131177
N
0
165
330
660 Kilometers

Figure S7.

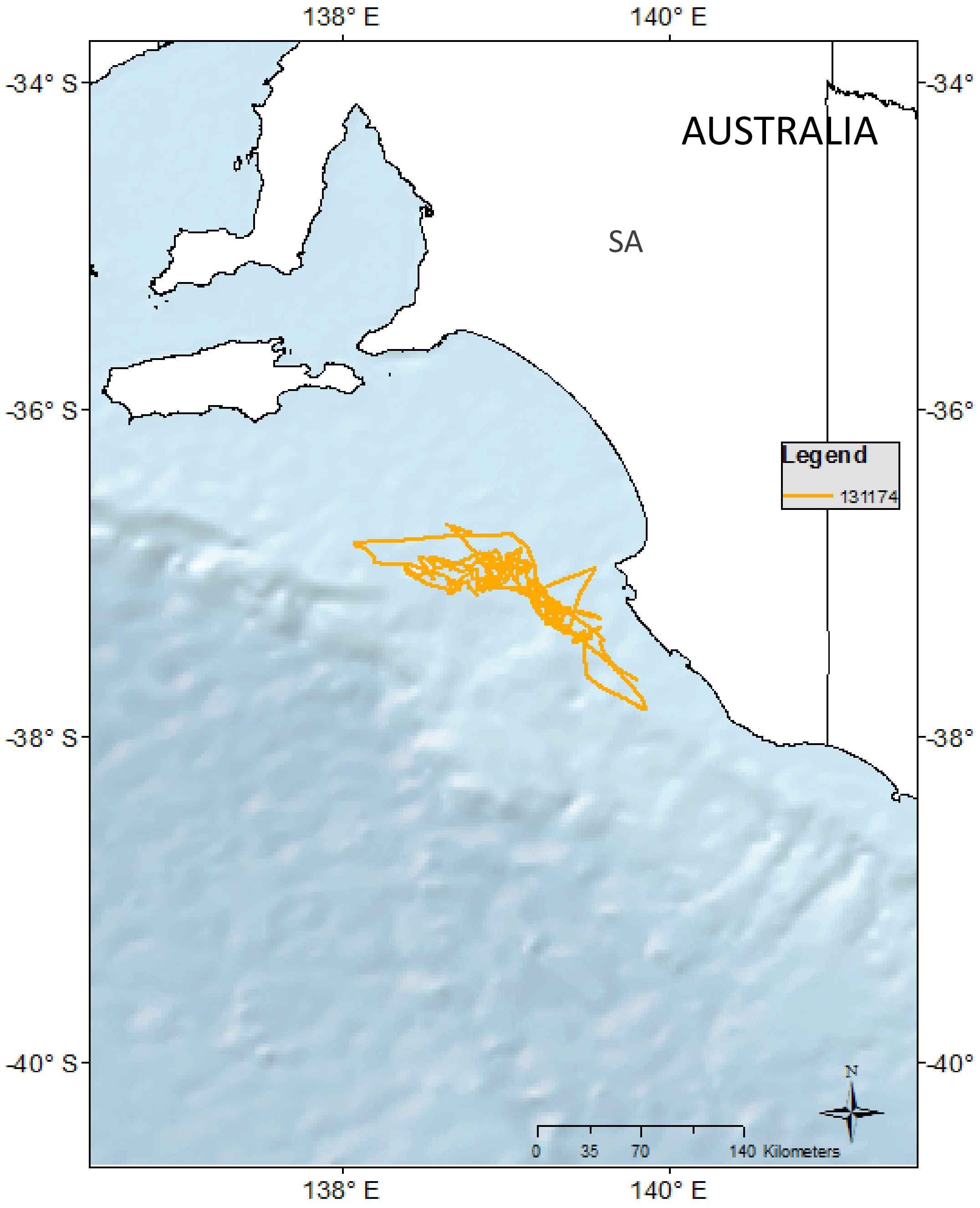

138° E
140° E
-34° S
-36° S
-38° S
-40° S
-34°
-36°
-38°
-40°
AUSTRALIA
SA
Legend
131174
N
0
35
70
140 Kilometers

Figure S8.

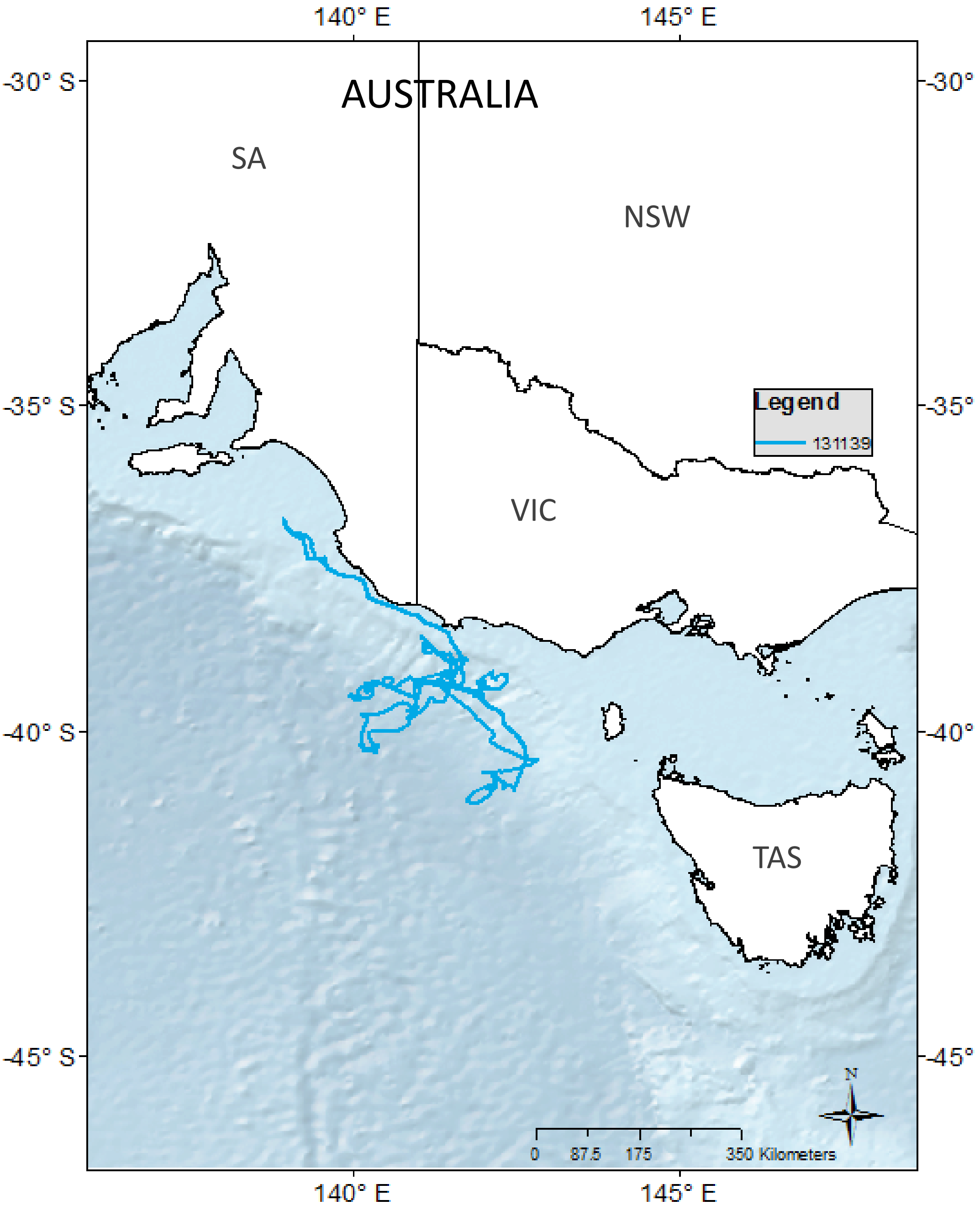
140° E
145° E
-30° S
-35° S
-40° S
-45° S
-30°
-35°
-40°
-45°
AUSTRALIA
SA
NSW
VIC
TAS
Legend
131139
N
0
87.5
175
350 Kilometers
140° E
145° E

Figure S9.

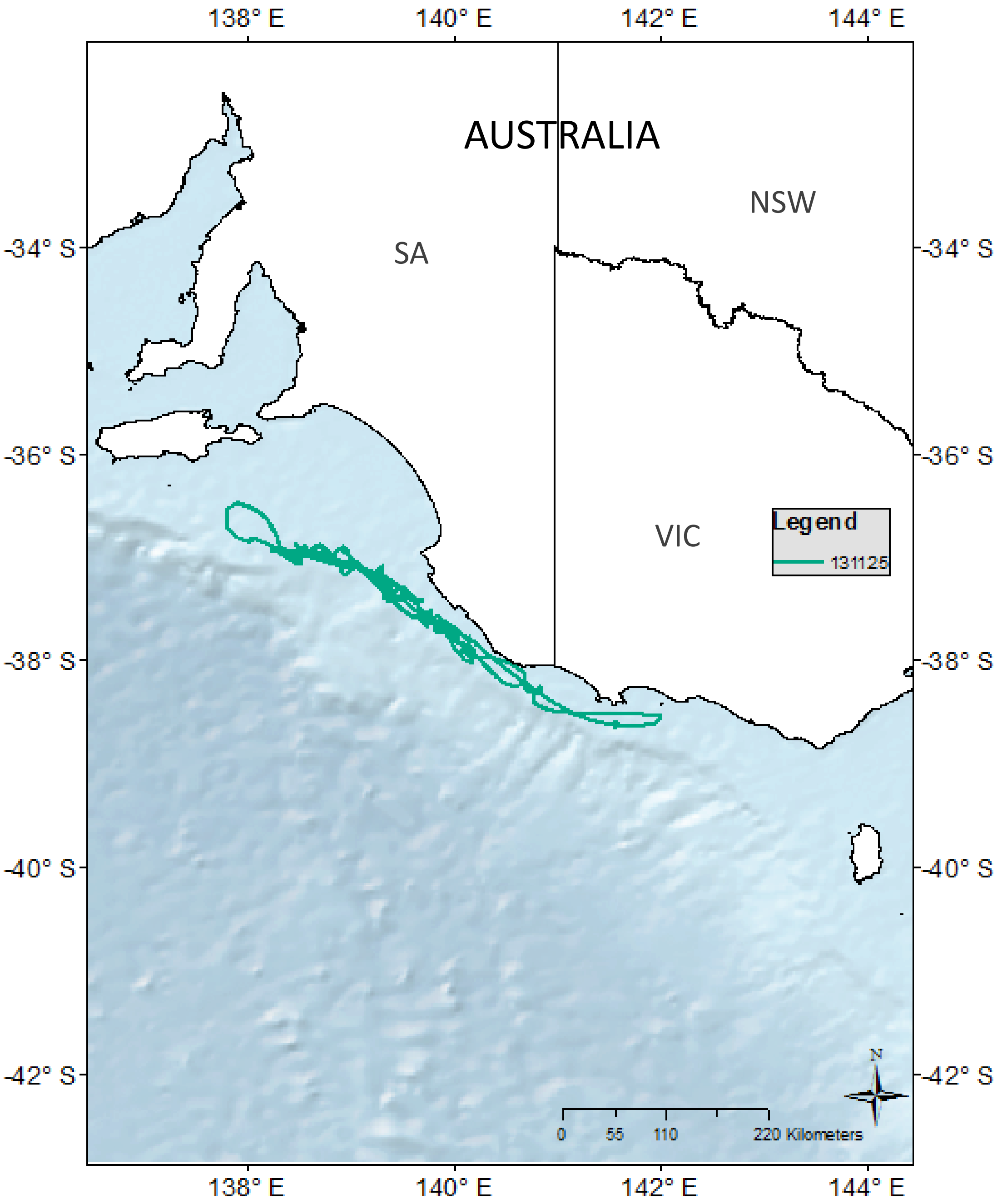
138° E
140° E
142° E
144° E
AUSTRALIA
NSW
SA
VIC
-34° S
-36° S
-38° S
-40° S
-42° S
Legend
131125
N
0
55
110
220 Kilometers

Figure S10.

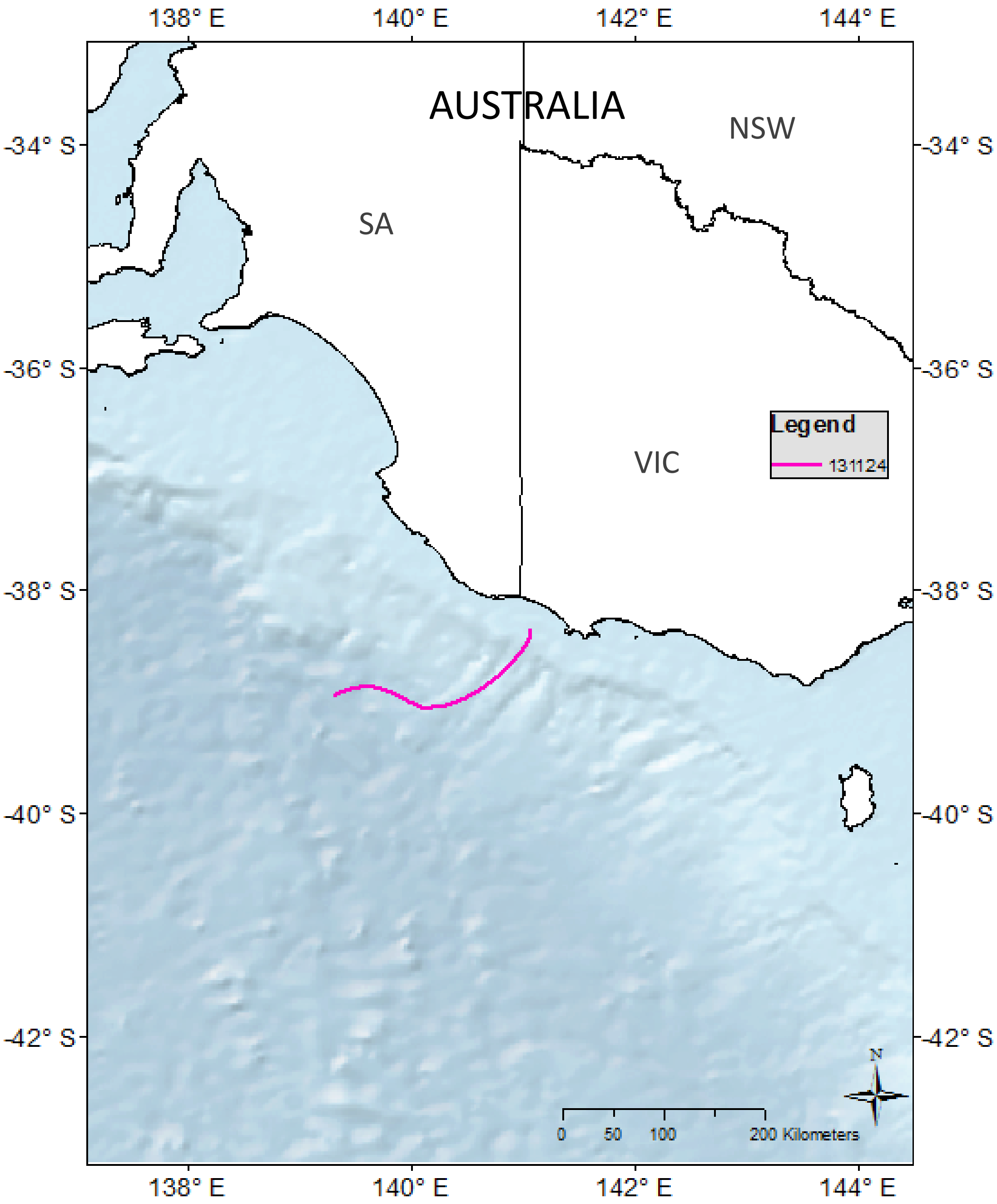

138° E
140° E
142° E
144° E
AUSTRALIA
NSW
SA
VIC
Legend
131124
-34° S
-36° S
-38° S
-40° S
-42° S
N
0
50
100
200 Kilometers

Figure S11.

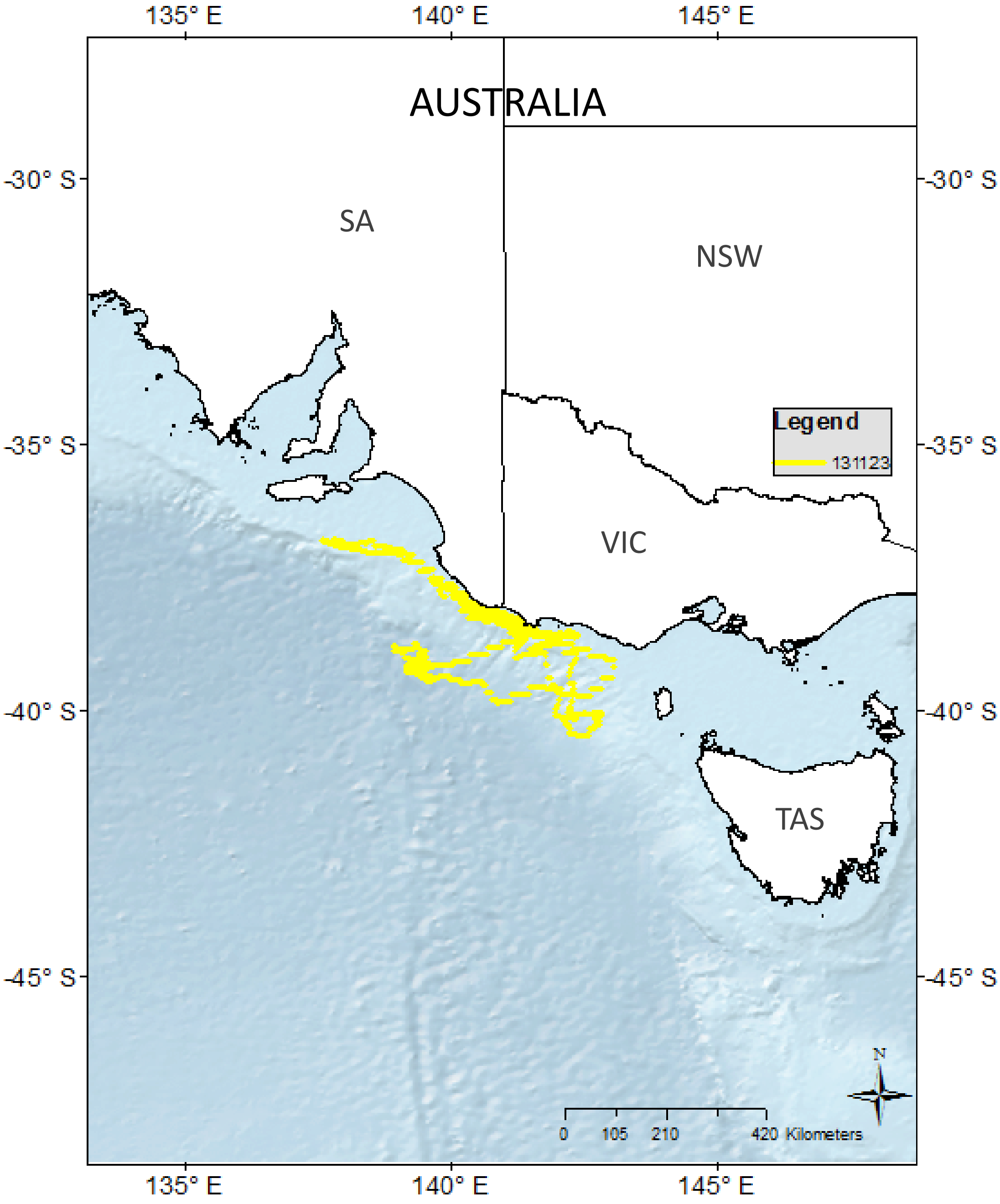

135° E
140° E
145° E
AUSTRALIA
-30° S
-35° S
-40° S
-45° S
SA
NSW
VIC
TAS
Legend
131123
0
105
210
420 Kilometers
N

Figure S12.

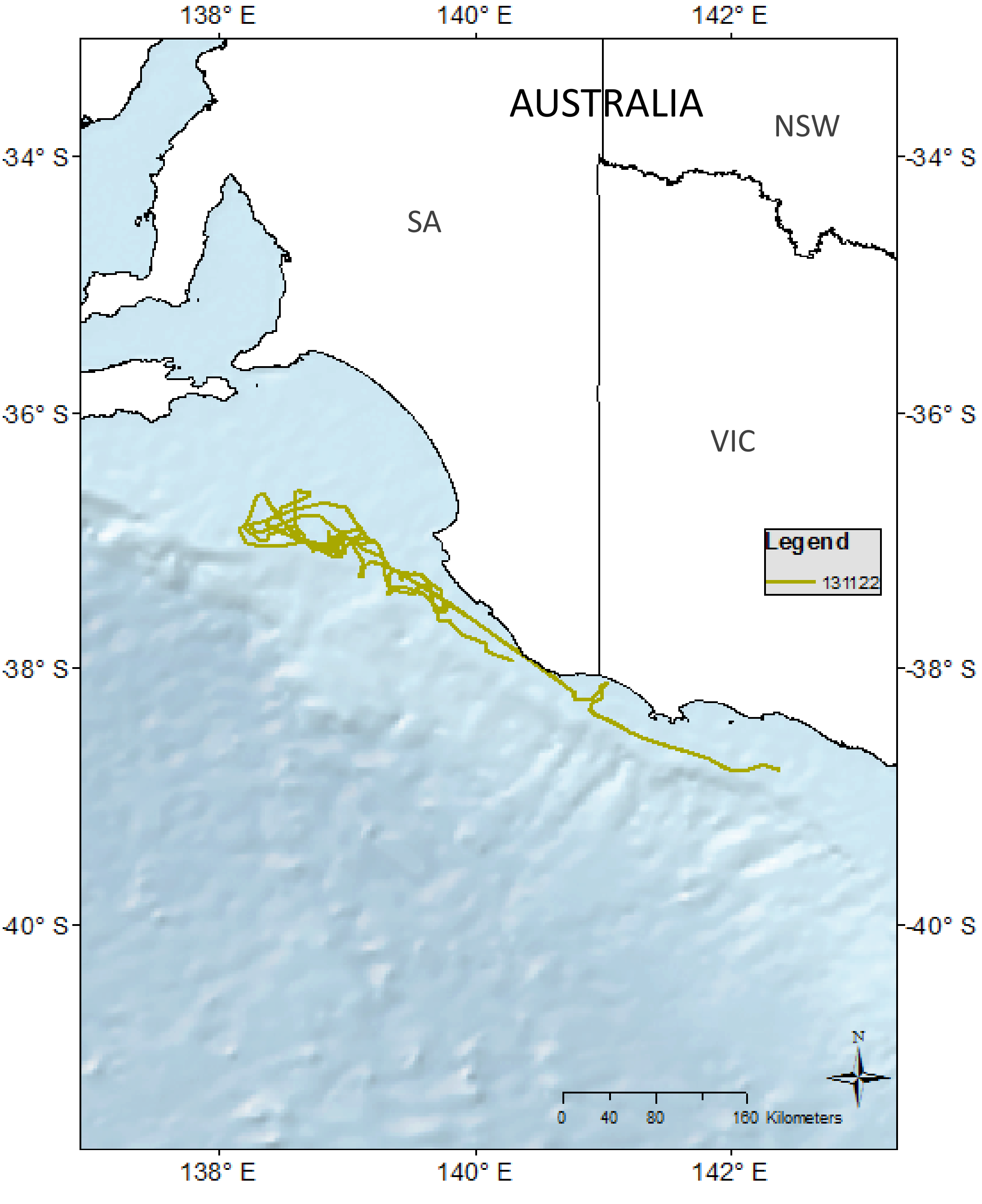
138° E
140° E
142° E
AUSTRALIA
NSW
SA
VIC
34° S
36° S
38° S
40° S
Legend
131122
N
0
40
80
160 Kilometers

Figure S13.

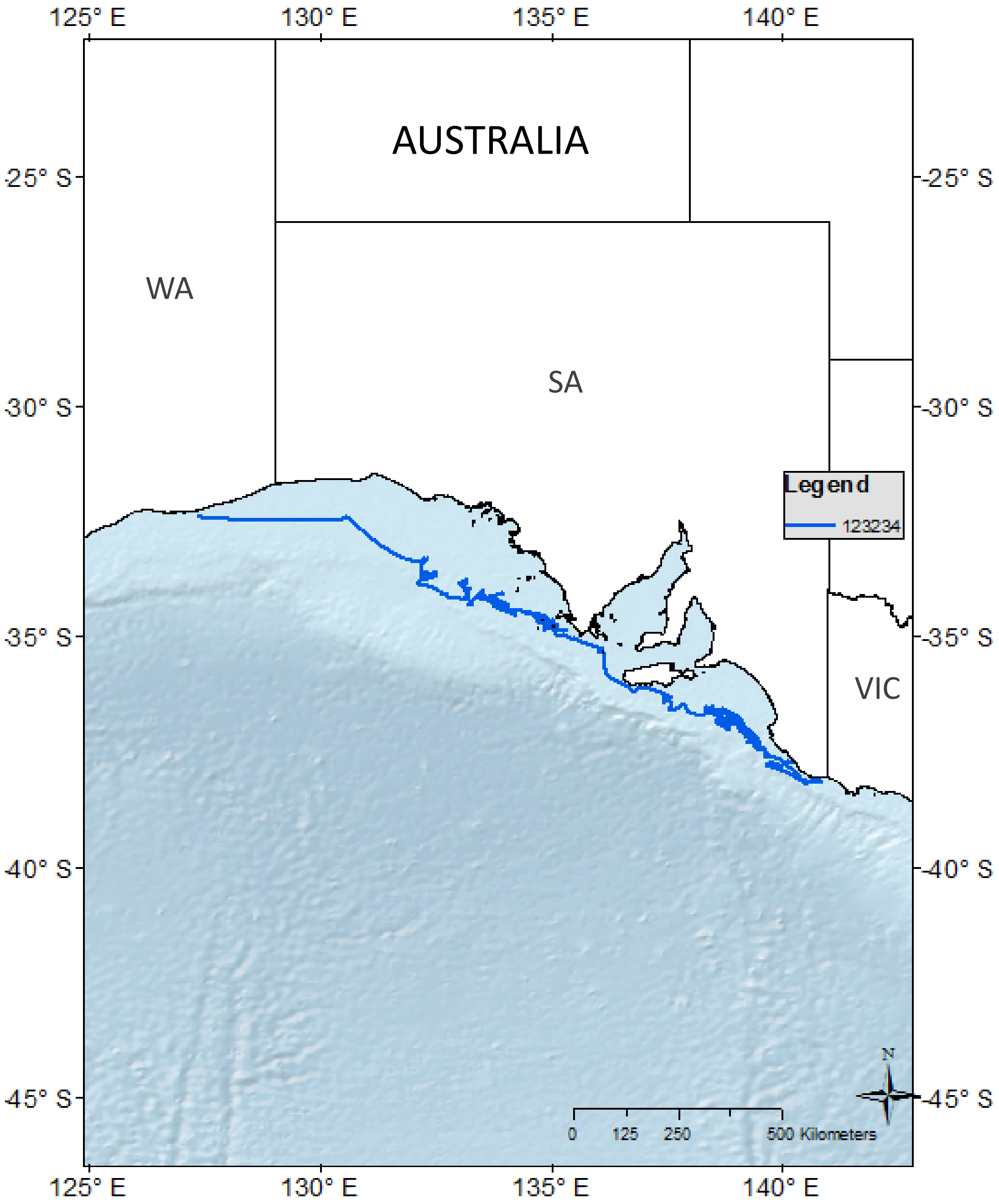
125° E
130° E
135° E
140° E
25° S
30° S
35° S
40° S
45° S
AUSTRALIA
WA
SA
VIC
Legend
123234
0
125
250
500 Kilometers
N

Figure S14.

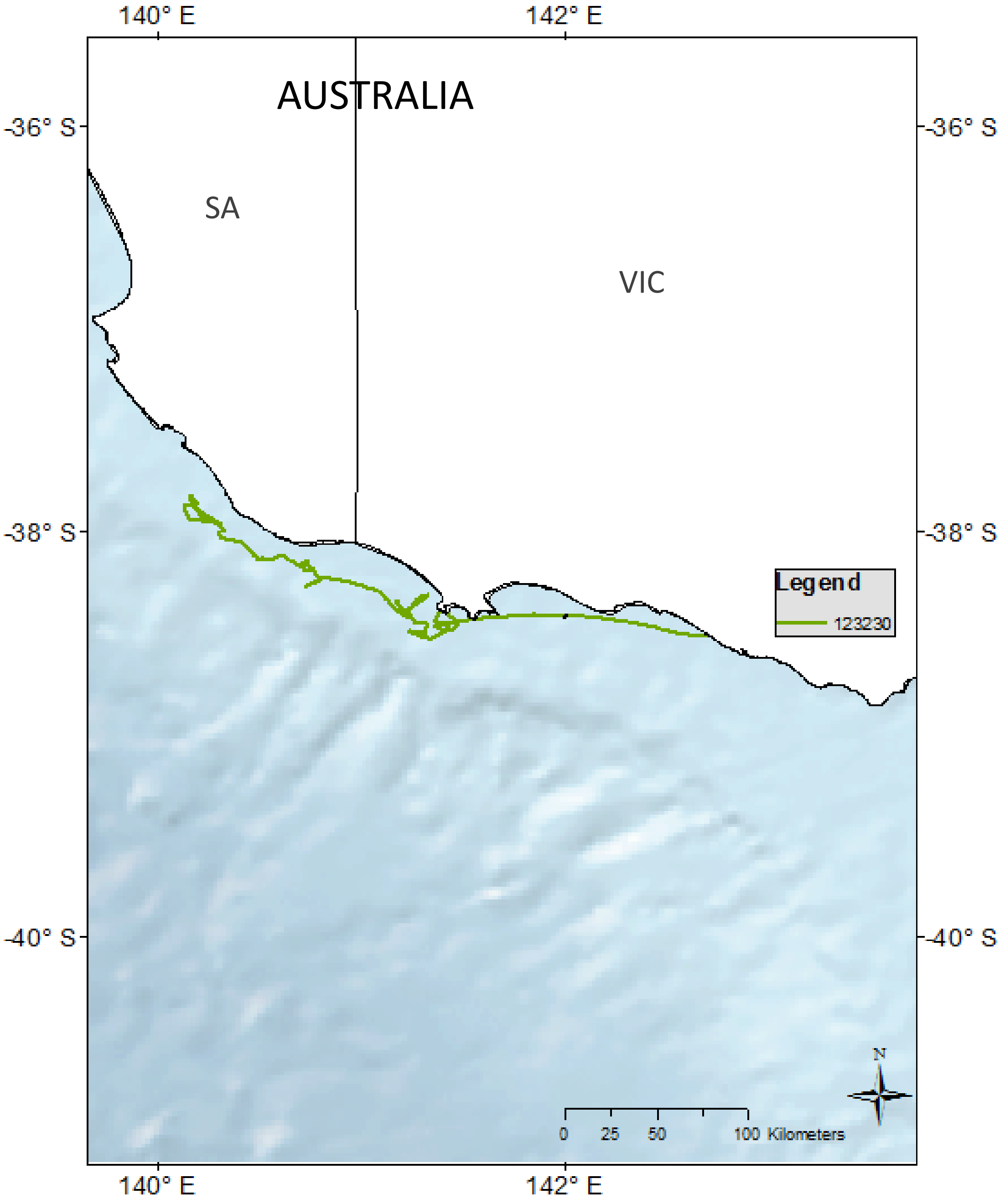
140° E
142° E
AUSTRALIA
-36° S
-38° S
-40° S
SA
VIC
Legend
123230
0
25
50
100 Kilometers
N

Figure S15.

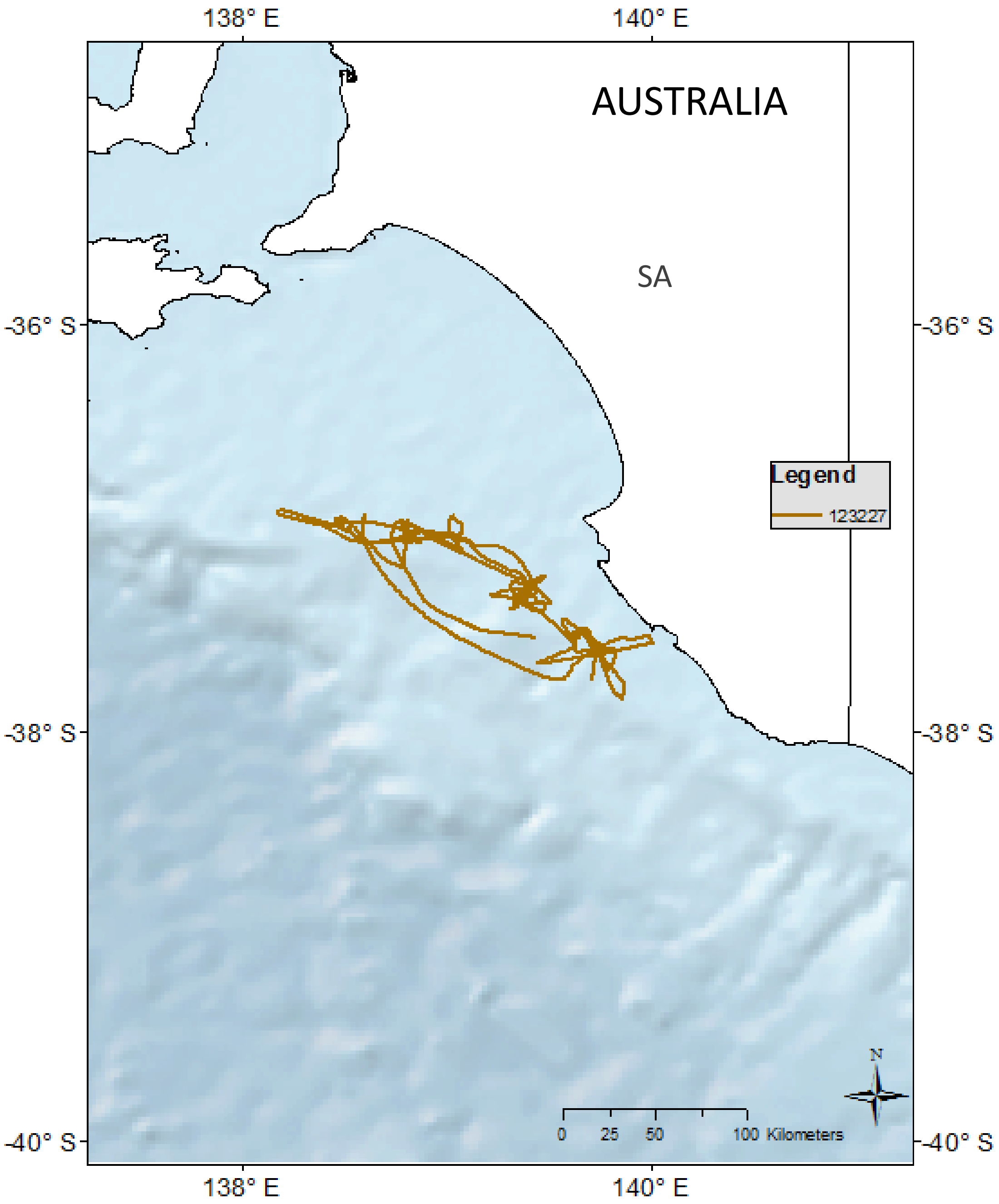

138° E
140° E
AUSTRALIA
SA
-36° S
-38° S
-40° S
Legend
123227
N
0 25 50 100 Kilometers

Table S1. (See excel spreadsheet)

Table S2. (See excel spreadsheet)
