# Supplementary material for: Movements and behaviour of blue whales satellite tagged in an Australian upwelling system
Source: Sci Rep. 2020 Dec 3;10:21165. doi: 10.1038/s41598-020-78143-2 (PMC7713308; doi:10.1038/s41598-020-78143-2)
Supplement: Supplementary file 4 — Supplementary Legends. [file 41598_2020_78143_MOESM4_ESM.docx]

**Supplementary figures**

Figure S1. Locations of 13 satellite tag deployments on pygmy blue whales (*Balaenoptera musculus brevicauda*) in the Bonney Upwelling region, Great Southern Australian Coastal Upwelling System, southern Australia, between January and March 2015. Deployment locations are marked with an ‘X’ and labelled with the tag numbers. SA = South Australia; VIC = Victoria. Map created in ArcGIS v.10 (available at <https://www.esri.com/>).

Figure S2. Clustering results of STRUCTURE analysis for 10 biopsy-sampled tagged blue whales (indicated in grey) with reference data of pygmy blue whales (*Balaenoptera musculus brevicauda*) and Antarctic blue whales (*B. m. intermedia*) (from Attard et al.^36,37^). Runs were summarised using CLUMPAK. Each individual is represented by a column.

Figure S3. Track of pygmy blue whale #123229. Map created in ArcGIS v.10 (available at <https://www.esri.com/>).

Figure S4. Track of pygmy blue whale #123233. Map created in ArcGIS v.10 (available at <https://www.esri.com/>).

Figure S5. Track of pygmy blue whale #123235. Map created in ArcGIS v.10 (available at <https://www.esri.com/>).

Figure S6. Track of pygmy blue whale #131177. Map created in ArcGIS v.10 (available at <https://www.esri.com/>).

Figure S7. Track of pygmy blue whale #131174. Map created in ArcGIS v.10 (available at <https://www.esri.com/>).

Figure S8. Track of pygmy blue whale #131139. Map created in ArcGIS v.10 (available at <https://www.esri.com/>).

Figure S9. Track of pygmy blue whale #131125. Map created in ArcGIS v.10 (available at <https://www.esri.com/>).

Figure S10. Track of pygmy blue whale #131124. Map created in ArcGIS v.10 (available at <https://www.esri.com/>).

Figure S11. Track of pygmy blue whale #131123. Map created in ArcGIS v.10 (available at <https://www.esri.com/>).

Figure S12. Track of pygmy blue whale #131122. Map created in ArcGIS v.10 (available at <https://www.esri.com/>).

Figure S13. Track of pygmy blue whale #123234. Map created in ArcGIS v.10 (available at <https://www.esri.com/>).

Figure S14. Track of pygmy blue whale #123230. Map created in ArcGIS v.10 (available at <https://www.esri.com/>).

Figure S15. Track of pygmy blue whale #123227. Map created in ArcGIS v.10 (available at <https://www.esri.com/>).

**Supplementary tables**

Table S1. Tracks of satellite tagged pygmy blue whales (*Balaenoptera musculus brevicauda*) used for the analysis in bsam (hSSSM). These were split into segments when gaps in transmission exceeded 48 hours. The day of tag deployment is displayed in black. Track sections for each whale are displayed in dark grey, with crosses representing additional days when the whale was in the Great Southern Australian Coastal Upwelling System (east of 125^o^E). Days in orange were excluded from the analysis due to short length of transmission.

Table S2. Environmental variables and associated datasets examined as predictors of pygmy blue whale (*Balaenoptera musculus brevicauda*) ARS behaviour in the Great Southern Australian Coastal Upwelling System (east of 125^o^E).

Table S1. (See excel spreadsheet)

Table S2. (See excel spreadsheet)
